# Platelet olfactory receptor activation limits platelet reactivity and growth of aortic aneurysms

**DOI:** 10.1172/JCI152373

**Published:** 2022-05-02

**Authors:** Craig N. Morrell, Doran Mix, Anu Aggarwal, Rohan Bhandari, Matthew Godwin, Phillip Owens, Sean P. Lyden, Adam Doyle, Krystin Krauel, Matthew T. Rondina, Amy Mohan, Charles J. Lowenstein, Sharon Shim, Shaun Stauffer, Vara Prasad Josyula, Sara K. Ture, David I. Yule, Larry E. Wagner, John M. Ashton, Ayman Elbadawi, Scott J. Cameron

**Affiliations:** 1 Aab Cardiovascular Research Institute, Department of Medicine, Microbiology and Immunology, and Laboratory Medicine and; 2 Department of Surgery, Division of Vascular Surgery, University of Rochester School of Medicine, New York, New York, USA.; 3 Department of Cardiovascular and Metabolic Sciences, Lerner Research Institute, Cleveland, Ohio, USA.; 4 Division of Cardiovascular Health and Disease, Department of Internal Medicine, University of Cincinnati College of Medicine, Cincinnati, Ohio, USA.; 5 Heart, Vascular and Thoracic Institute, Department of Vascular Surgery, Cleveland, Ohio, USA.; 6 Departments of Internal Medicine and Pathology and the Molecular Medicine Program at the University of Utah Health Sciences Center, Eccles Institute of Human Genetics, Salt Lake City, Utah, USA.; 7 Department of Internal Medicine and GRECC at the George E. Wahlen VAMC, Salt Lake City, Utah, USA.; 8 Molecular Medicine Program, University of Utah, Salt Lake City, Utah, USA.; 9 Department of Cardiology and Angiology I, Heart Center, University of Freiburg, Freiburg, Germany.; 10 Department of Medicine, Division of Cardiology, Johns Hopkins University School of Medicine, Baltimore, Maryland, USA.; 11 Cleveland Clinic Center for Therapeutics Discovery, Lerner Research Institute, Cleveland, Ohio, USA.; 12 Department of Pharmacology and Physiology and; 13 Department of Biomedical Genetics, University of Rochester School of Medicine, Rochester, New York, USA.; 14 Department of Cardiovascular Medicine, University of Texas Medical Branch, Galveston, Texas, USA.; 15 Heart Vascular and Thoracic Institute, Department of Cardiovascular Medicine, Section of Vascular Medicine, Cleveland Clinic Foundation, Cleveland, Ohio, USA.; 16 Case Western Reserve University Lerner College of Medicine, Cleveland, Ohio, USA.; 17 Department of Hematology, Taussig Cancer Center, Cleveland, Ohio, USA.

**Keywords:** Vascular Biology, Platelets, Signal transduction, Thrombosis

## Abstract

As blood transitions from steady laminar flow (S-flow) in healthy arteries to disturbed flow (D-flow) in aneurysmal arteries, platelets are subjected to external forces. Biomechanical platelet activation is incompletely understood and is a potential mechanism behind antiplatelet medication resistance. Although it has been demonstrated that antiplatelet drugs suppress the growth of abdominal aortic aneurysms (AAA) in patients, we found that a certain degree of platelet reactivity persisted in spite of aspirin therapy, urging us to consider additional antiplatelet therapeutic targets. Transcriptomic profiling of platelets from patients with AAA revealed upregulation of a signal transduction pathway common to olfactory receptors, and this was explored as a mediator of AAA progression. Healthy platelets subjected to D-flow ex vivo, platelets from patients with AAA, and platelets in murine models of AAA demonstrated increased membrane olfactory receptor 2L13 (OR2L13) expression. A drug screen identified a molecule activating platelet OR2L13, which limited both biochemical and biomechanical platelet activation as well as AAA growth. This observation was further supported by selective deletion of the OR2L13 ortholog in a murine model of AAA that accelerated aortic aneurysm growth and rupture. These studies revealed that olfactory receptors regulate platelet activation in AAA and aneurysmal progression through platelet-derived mediators of aortic remodeling.

## Graphical abstract

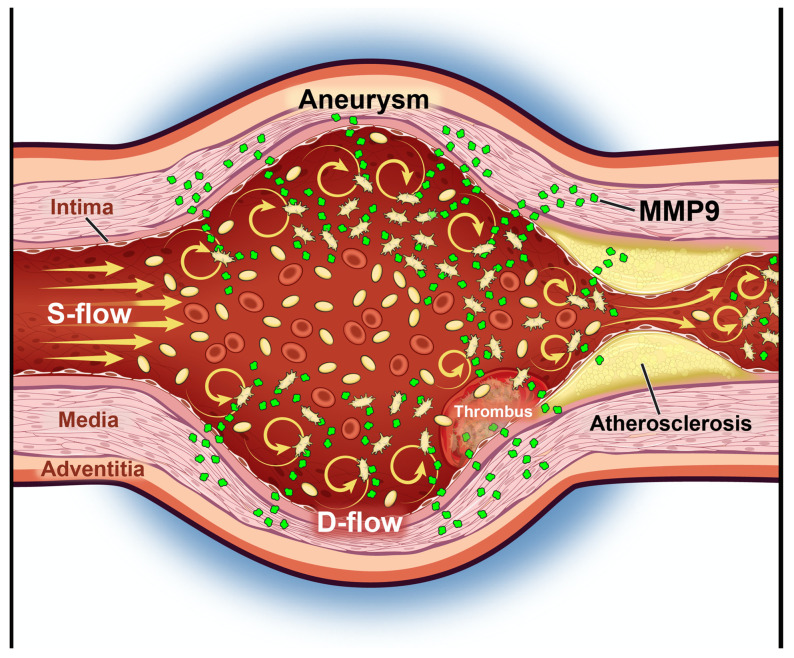

## Introduction

Remodeling of the infrarenal aorta that results in abdominal aortic aneurysms (AAA) affects millions of individuals and carries an extraordinarily high out-of-hospital mortality rate if this pathological process progresses to aortic rupture (1). Most individuals with AAA are asymptomatic but harbor common risk factors, including male sex, tobacco use, advanced age, and atherosclerosis. Factors accelerating AAA growth involve mechanical forces on the blood vessel wall and enzymatic degradation of the extracellular matrix (ECM) (2).

Platelet aggregates and thrombi are found in aneurysmal arterial segments of patients with infrarenal AAA, raising the possibility that platelets play a role in the etiology of AAA (3–6). Unlike healthy platelets, circulating platelets in humans and in murine models of cardiovascular disease are enriched in activated MMPs and if secreted, MMPs may increase the vulnerability of arteries to aneurysm development and rupture (7–9). In a murine model of AAA, platelet antagonists were shown to inhibit AAA rupture coincident with inhibition of aortic MMP activity (4). The mechanism for this protective effect of platelet antagonists was not determined. Supporting this observation, we recently interrogated an administrative database of several million patients, revealing that long-term antiplatelet medication use was a highly significant factor that confers protection from AAA development, dissection, and rupture (10).

As blood transitions from steady laminar flow (S-flow) to disturbed flow (D-flow) in aneurysmal arteries, circulating platelets may become mechanically activated (11). Mechanical platelet activation in patients with aneurysmal arteries was postulated using computational fluid dynamic modeling. Agonist-mediated platelet activation has never been proven in humans with infrarenal AAA or in relevant animal models of AAA (6). Subjecting blood to mechanical forces promotes the development of luminal thrombus. Luminal thrombus was suggested to accelerate aneurysmal growth of arteries (12, 13). In a recent randomized clinical trial in patients with small AAA, treatment with the platelet P2Y_12_ receptor antagonist ticagrelor did not alter AAA growth, indicating a more mechanistic approach to understanding platelet activation in AAA is required (14).

Presently available therapeutic agents to abrogate platelet reactivity focus on cell surface receptors initiating biochemical second messenger–mediated signaling cascades. There are no therapeutic agents to inhibit biomechanical platelet activation. This investigation sought to characterize platelets from patients with infrarenal AAA to identify mechanisms by which platelets may be involved in the etiology of AAA through exposure to external forces in aneurysmal arteries. Our investigation revealed a therapeutic target made available on the surface of biomechanically activated platelets belonging to the olfactory receptor family of GPCRs. This olfactory receptor was evaluated as a mediator of biochemical signaling in platelets that may regulate AAA progression, thus highlighting a new platelet target for treating vascular disorders.

## Results

### Platelets from patients with AAA are highly reactive and enriched in activated MMPs.

**Figure 1:**
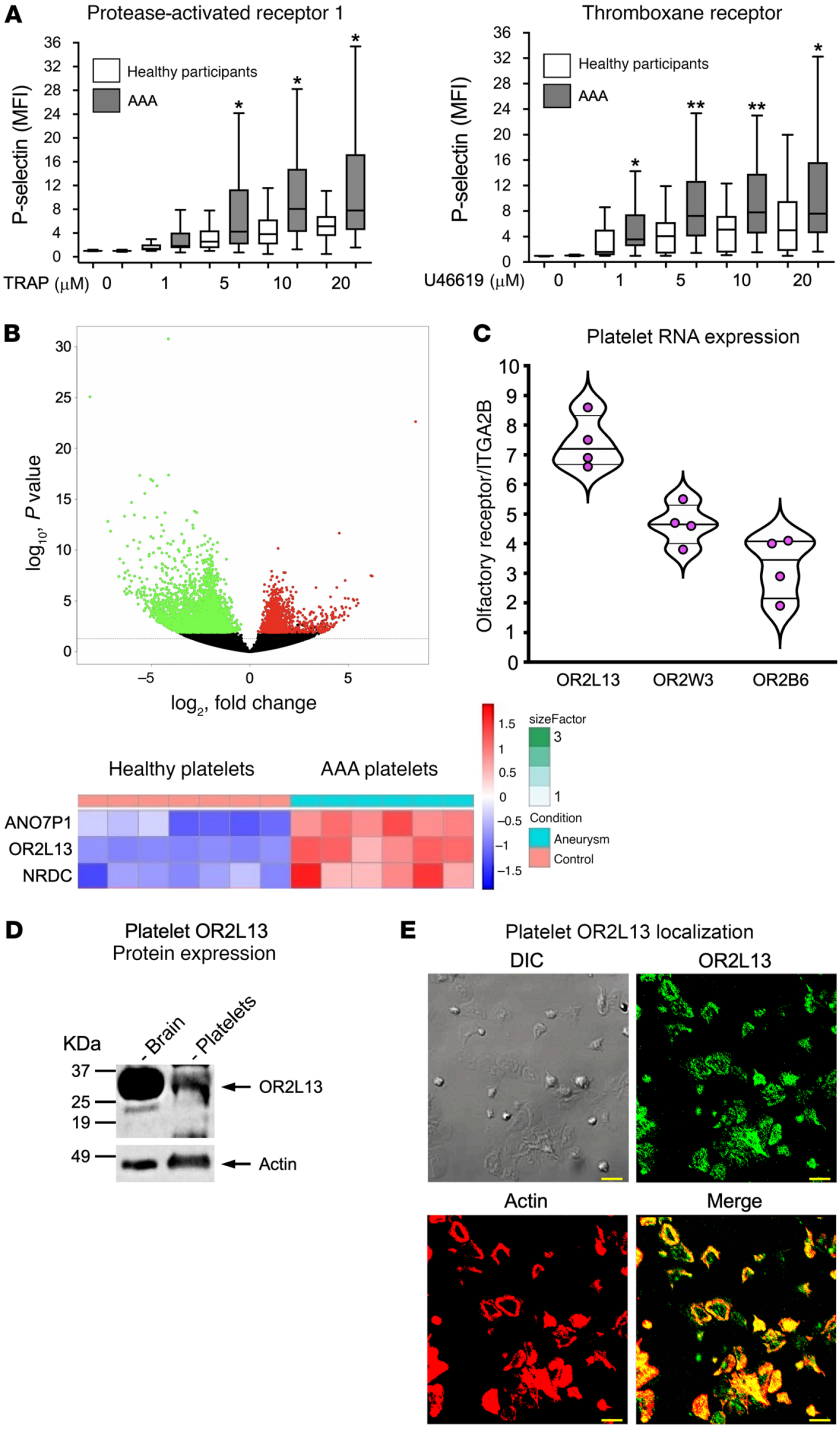
Platelet reactivity is enhanced in patients with AAA. (**A**) Washed platelets from patients with AAA (*n =* 18) compared with healthy individuals (*n =* 10). Platelet activation before and after stimulation with a thromboxane receptor agonist (U46619) or a PAR1 agonist (TRAP-6) for 15 minutes. Platelet activation was quantified by FACS as the MFI and is represented as the median (horizontal line) in a box-and-whisker plot for each group, performed in quadruplicate and summed for each patient at each concentration of agonist. *P < 0.05 and **P < 0.01 versus healthy control; group differences were analyzed by Kruskal-Wallis test followed by Dunn’s post test correction. (**B**) Human platelet RNA was extracted from healthy individuals (*n =* 7) and compared with that of patients with AAA (*n =* 6) by mRNA-Seq. Volcano plot shows genes upregulated (red) and downregulated (green) in patients with AAA. Dashed line is the threshold of discrimination. Heatmap with upstream olfactory receptor 2L13 (OR2L13) and downstream anoctamin, which was higher in AAA platelets compared with heathy conditions. (**C**) RNA isolated from washed platelets followed by CD45-mediated immunodepletion of WBCs; then qRT-PCR normalized to the platelet GPIIb gene (ITGA2B). Data from 4 individual healthy males, each run in quadruplicate, are indicated by violin plots for the 3 olfactory receptors present in every individual tested. (**D**) Lysate from healthy human platelets or human brain (positive control) separated by SDS-PAGE before probing with an anti-OR2L13 antibody. (**E**) Platelets from healthy humans on a fibrinogen matrix assessed for OR2L13 immunofluorescence by confocal microscopy. A FITC-tagged OR2L13 antibody (green) and rhodamine-tagged phalloidin (red) for filamentous actin. DIC, differential interference contrast. Scale bars: 10 μm.

To determine whether platelets are more active in patients with AAA, we isolated washed platelets from patients or from healthy individuals and stimulated them with surface receptor agonists. Despite daily antiplatelet therapy for the majority of patients with AAA (Supplemental Figure 1; supplemental material available online with this article; https://doi.org/10.1172/JCI152373DS1), platelet reactivity through both the thromboxane receptor and PAR1 was increased (Figure 1A). Platelet hyperreactivity was not observed by stimulating the P2Y_12_ receptor (Supplemental Figure 2). Platelet surface PAR1 and thromboxane receptor density were not significantly different in patients with AAA compared with healthy control platelets (Supplemental Figure 3). These findings suggest that circulating platelets in patients with AAA are phenotypically different.

### Olfactory signaling proteins are increased in platelets from patients with AAA.

**Figure 2:**
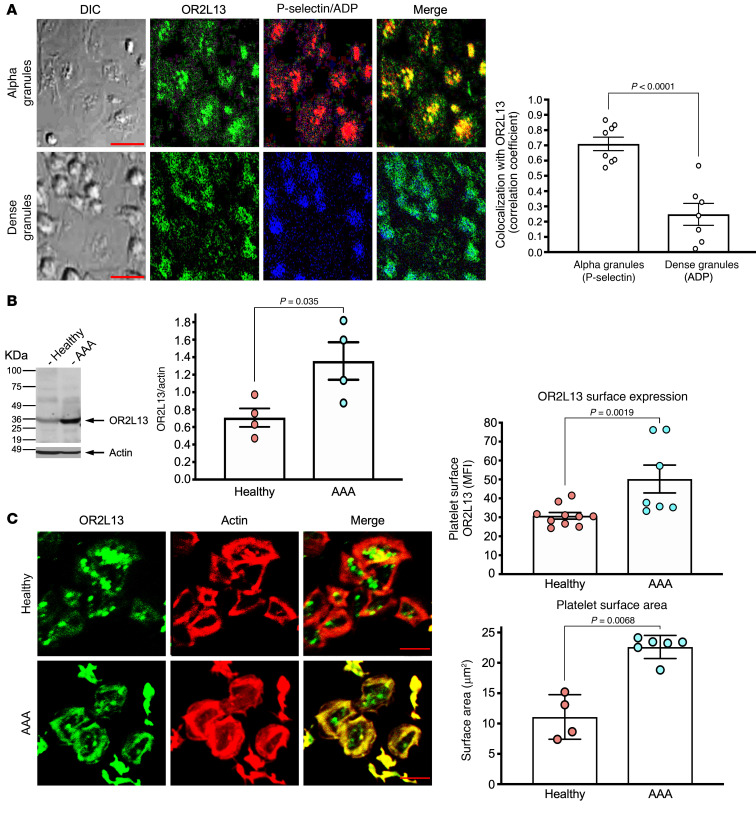
Platelet olfactory receptor expression increases in AAA. (**A**) Alpha granules were identified by a rhodamine-tagged P selectin antibody (red). Dense granules were detected by staining phosphate groups in adenosine diphosphate (ADP, blue) by confocal microscopy. Spearman’s ρ was determined by computer-generated colocalization overlay of OR2L13 and P selectin (0.71 ± 0.04) or ADP (0.25 ± 0.07) and is represented as the mean ± SEM. *n =* 7–8 individuals. *P* < 0.0001, by 2-tailed Student’s *t* test. Scale bars: 10 μm. (**B**) Immunoblotting platelets for OR2L13 expression, which was increased in AAA compared with healthy conditions, is represented as the mean ± SEM. *n =* 4 in each group. *P =* 0.035, by 2-tailed Student’s *t* test. (**C**) OR2L13 localization and surface area by confocal microscopy and flow cytometry. Confocal microscopy was used to visualize platelet surface area by spreading on a fibrinogen matrix, with quantification as the mean surface area ± SEM. *n =* 7–10. Mann-Whitney *U* test. Scale bars: 5 μm. Platelet surface OR2L13 was quantified by FACS as the MFI ± SEM. *n =* 4–6. Mann-Whitney *U* test.

In order to determine platelet signaling pathways that may be altered in AAA, we isolated platelet RNA from healthy individuals and from patients with AAA and then performed RNA-Seq (Supplemental Figure 4). Differential expression of multiple transcripts was found between AAA and healthy control platelets, with the top 2 upregulated transcripts encoding OR2L13 and anoctamin 7, which are components of olfactory receptor signal transduction (Figure 1B). More than 400 olfactory genes exist in the human genome (15). A functional role for platelet olfactory receptors has not been previously reported. We validated this primary observation in platelets using several complementary techniques. By employing RNA-Seq in human CD34+, cord-blood derived, cultured megakaryocytes (platelet precursors), we examined which olfactory genes are endogenously expressed. We identified 15 nontruncated olfactory receptor transcripts (including OR2L13) in human megakaryocytes (Supplemental Figure 5A). We validated the expression of only a subset of olfactory receptor transcripts in twice-washed, CD45-depleted platelets from healthy adults by quantitative reverse transcriptase PCR (qRT-PCR) (Figure 1C and Supplemental Figure 5B). Several olfactory receptor pseudogenes with unclear function were also detected. Just 3 mature olfactory receptors were detected in adult platelets from every healthy individual tested: OR2L13, OR2W3, and OR2B6 (Figure 1C). Transcripts found only in some adult platelets are shown in Supplemental Figure 5B. NRDC (nardilysin convertase), a required enzyme recently reported for platelet budding from megakaryocytes under D-flow conditions (16), was upregulated in platelets from patients with AAA by RNA-Seq and found to be present in healthy platelets by qRT-PCR (Supplemental Figure 5B). Using human brain lysate as a positive control, healthy platelets were confirmed to express full-length OR2L13 protein by immunoblotting, and OR2L13 membrane expression was observed by confocal microscopy (Figure 1, D and E). By co-staining platelet alpha granule and dense granule markers with OR2L13, coexpression of OR2L13 primarily with P selectin suggested alpha granule storage and trafficking to the plasma membrane (Figure 2A). Because OR2L13 was the only olfactory receptor for which expression in platelets changed in AAA, we focused on understanding OR2L13 signal transduction. Protein expression of OR2L13 and anoctamin 7 was increased in platelets from patients with AAA compared with healthy controls (Figure 2B and Supplemental Figure 5C). Sex-dependent differences in platelet function were previously reported (7, 17, 18). However, we found platelet olfactory receptors were expressed similarly in healthy men and women (not shown). Membrane OR2L13 expression was increased in platelets from patients with AAA, and AAA platelet spreading (surface area) on a fibrinogen matrix was also greater, further confirming enhanced platelet reactivity in AAA through the glycoprotein IIb/IIa (GPIIb/IIIa) receptor (Figure 2C).

### OR2L13 is biomechanically sensitive.

**Figure 3:**
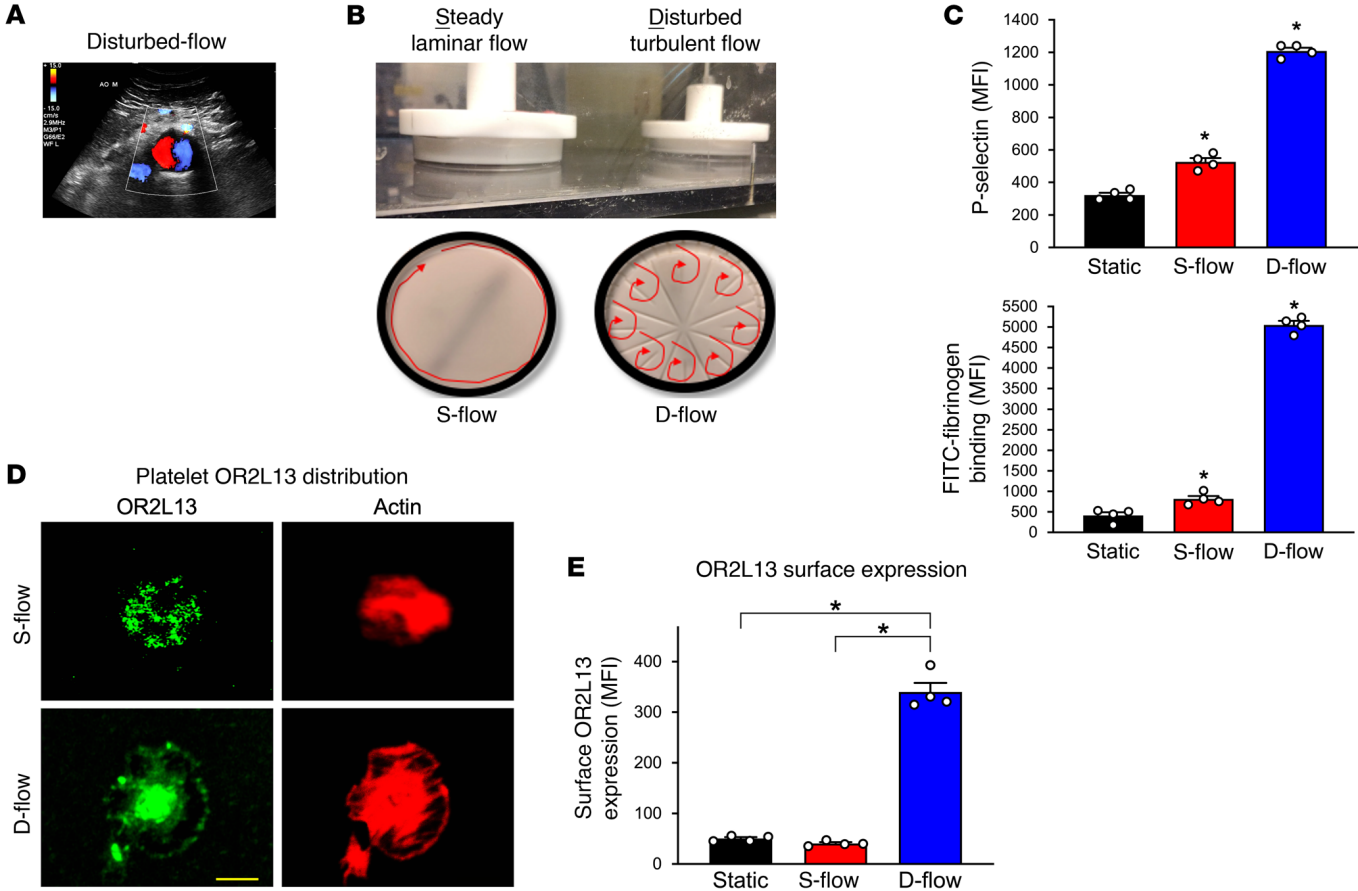
Biomechanical stimulation of platelets increases activation. (**A**) Color flow Doppler imaging of a large infrarenal AAA demonstrates an alternating direction of blood flow (disturbed flow, D-flow). (**B**) An in vitro flow-and-cone system was used to subject healthy platelets to steady laminar flow (S-flow) or D-flow for 120 minutes. (**C**) Platelet activation following S-flow and D-flow was quantified by surface P selectin expression or fibrinogen binding by FACS as the MFI ± SEM. *n =* 4. *P <* 0.001 versus static flow, by 1-way ANOVA followed by Bonferroni’s correction. (**D**) Healthy human platelets were subjected to static flow, S-flow, or D-flow for 120 minutes. Immobilization of platelets after S-flow and D-flow on a fibrinogen matrix and visualization by confocal microscopy. Platelets were stained with an FITC-tagged antibody for OR2L13 (green) and rhodamine-tagged phalloidin for actin (red). Scale bar: 5 μm. (**E**) In a separate set of experiments, platelet membrane OR2L13 was quantified after S-flow and D-flow by FACS as the MFI ± SEM. *n =* 4. *P <* 0.001 versus static flow, by 1-way ANOVA followed by Bonferroni’s correction.

Patients with AAA have an aorta with an irregular shape, exposing circulating platelets to D-flow, as noted by color spectral Doppler imaging (Figure 3A). An ex vivo flow-and-cone system was used to recapitulate exposure of platelets to S-flow and D-flow (ref. 19 and Figure 3B). D-flow was an especially potent stimulus for biomechanical platelet activation (Figure 3C), with OR2L13 converging in a central granulomere and on the membrane surface of permeabilized platelets (Figure 3D). A marked increase in platelet membrane OR2L13 distribution was confirmed after nonpermeabilized platelets were exposed to D-flow but not S-flow (Figure 3E, flow cytometry), suggesting the nature of biomechanical platelet activation is a trigger for OR2L13 translocation. Since platelets can synthesize and degrade proteins in response to environmental stressors (20, 21), we assessed OR2L13 protein expression after S-flow and D-flow exposure and found it to be similar to static conditions (not shown).

### OR2L13 ligands and platelet function.

**Figure 4:**
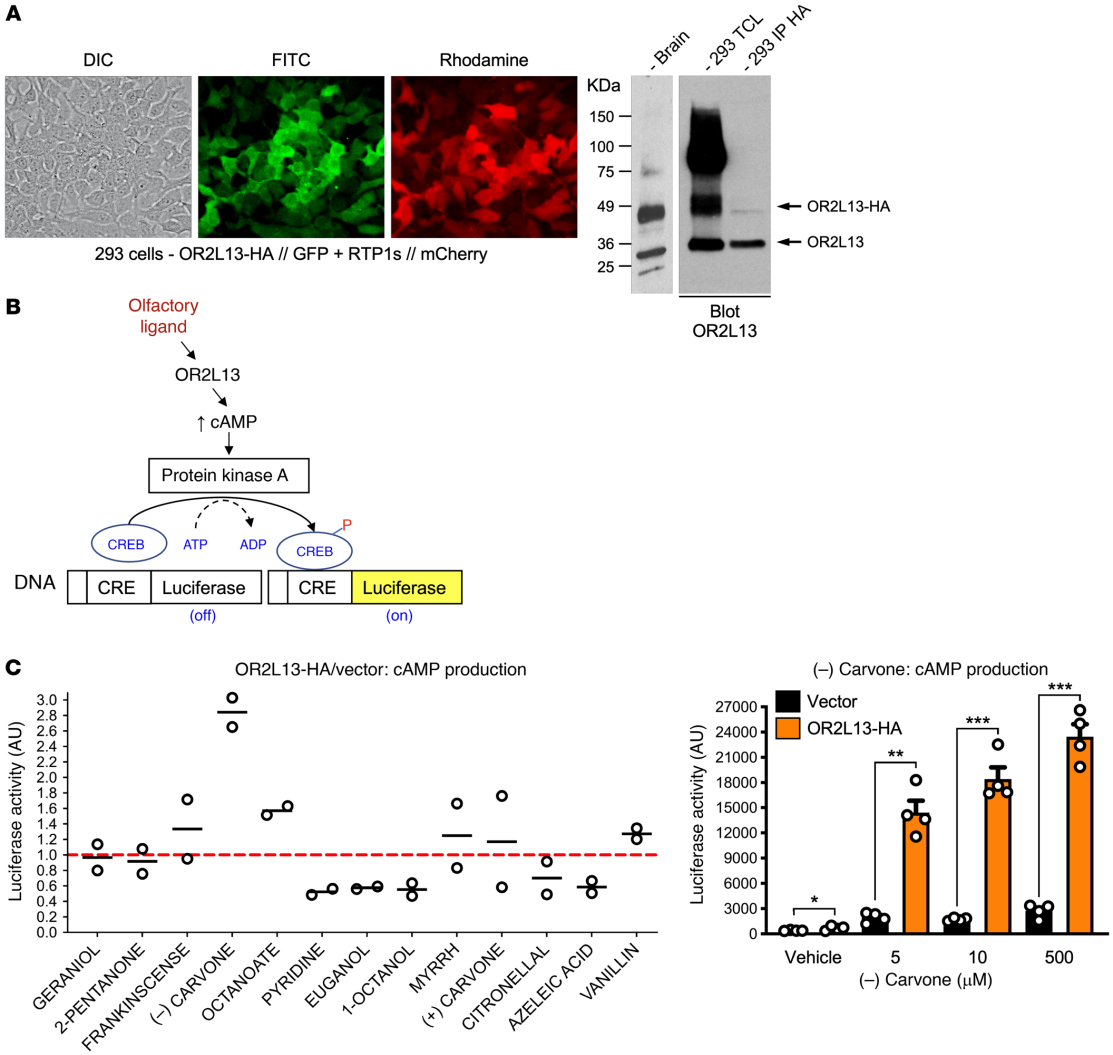
Characterization of OR2L13 agonists. (**A**) Human OR2L13 was subcloned in frame with HA (GFP in a secondary cassette, green) and coexpressed with receptor transport protein 1s (RTP1s) (mCherry in a secondary cassette, red) stably in HEK293/cAMP cells. Microscopy and Western blotting confirmed OR2L13-HA expression. Original magnification, ×20. TCL, total cell lysate. OR2L13 ligands stimulated (G_olf_), and adenyl cyclase produced cAMP. (**B**) A cAMP response element (CRE) expressing HEK293 cells with stable integration of OR2L13-HA was utilized to screen for ligands. (**C**) Alpha screen of olfactory ligands in OR2L13 transduced/nontransduced cells with more than 1.0 ratio (red dashed line) for OR2L13 activation; (–) carvone activated OR2L13 to generate cAMP (performed in duplicate, horizontal line indicates the mean). Results of a confirmatory experiment with vehicle versus (–) carvone are shown as the mean ± SEM. *n =* 4 (right). *P = 0.079, **P = 0.0001, and ***P < 0.0001 versus vehicle, by 1-way ANOVA followed by Bonferroni’s correction.

After olfactory receptor ligation by an external agonist, G_olf_ activates adenylyl cyclase to hydrolyze cAMP from ATP. We developed a HEK293 reporter cell line to rapidly evaluate potential OR2L13 odorant ligands based on postreceptor cAMP production. Human OR2L13 cDNA was cloned in a bicistronic vector with a gene encoding the chaperone protein receptor transport protein 1 subunit (RTP1s) to allow for efficient membrane localization (22). Lentivirus was stably transduced OR2L13 and RTP1s in HEK293 cells with a cAMP response element in the 5′ position in-frame with luciferase as a biological reporter (Figure 4, A and B). Using cells expressing only the empty vector as a control, multiple potential olfactory receptor ligands were evaluated. The terpene derivative (–) carvone reproducibly activated OR2L13, generating endogenous cAMP in a dose-dependent manner compared with forskolin as a positive control for endogenous adenylyl cyclase activation (Figure 4C and Supplemental Figure 6).

**Figure 5:**
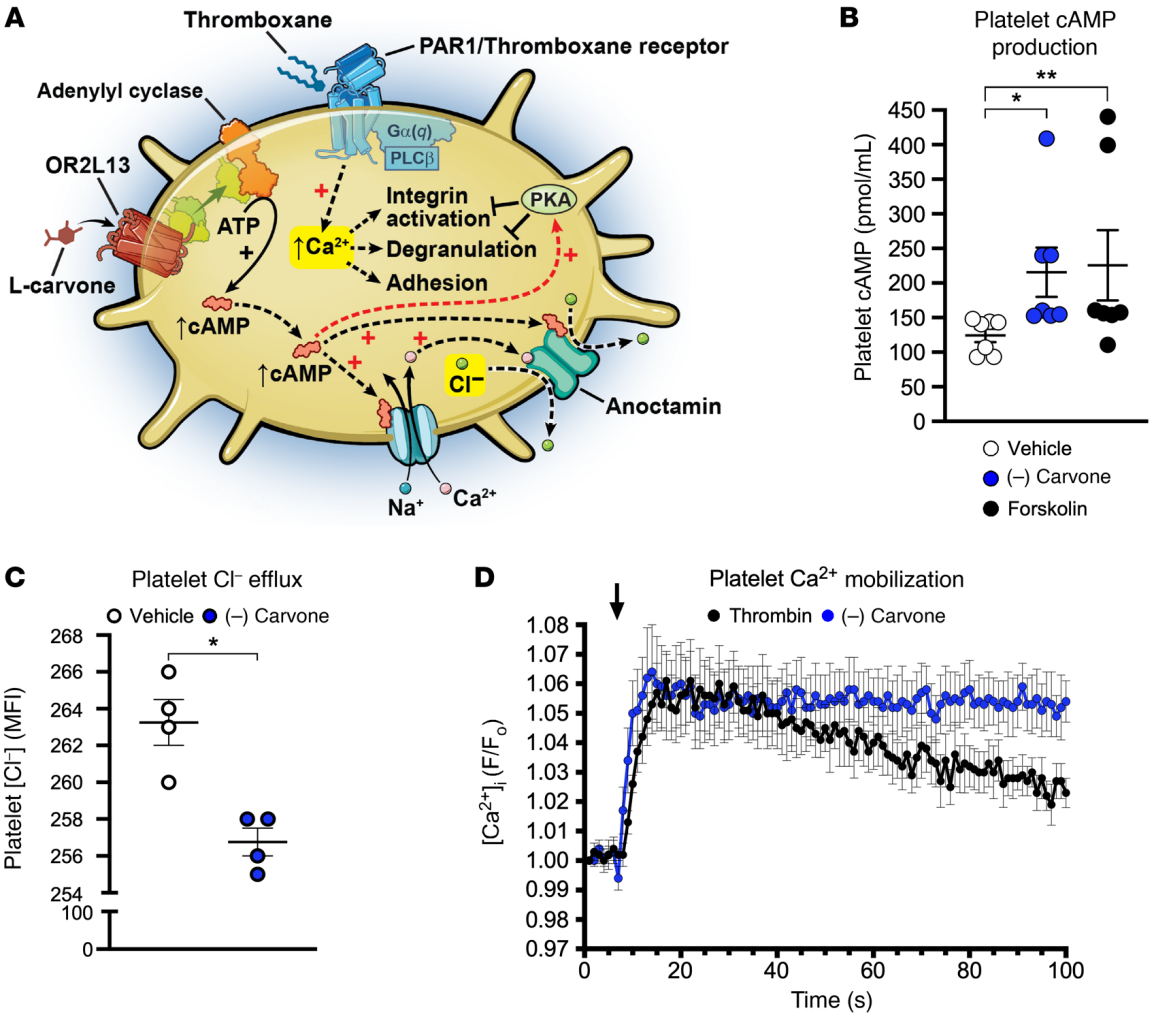
A conserved olfactory receptor signal transduction pathway in healthy platelets. (**A**) Proposed platelet OR2L13 signal transduction. Levo-carvone (L-carvone) binds to OR2L13 on platelets to generate cAMP through adenylyl cyclase activation, and cAMP changes the cytosolic concentration of calcium (Ca^2+^) directly and chloride conductance (Cl^–^), while inhibiting platelet reactivity through known, well-described mechanisms involving protein kinase A (PKA). (**B**) (–) Carvone (300 μM) or forskolin (10 μM) incubation with human platelets for 5 minutes generates cAMP. Forskolin was used as a positive control for adenylyl cyclase activity and ATP hydrolysis to cAMP. *n =* 7. *P = 0.0059 and **P = 0.0089 versus vehicle, by Kruskal-Wallis test followed by Dunn’s post test. (**C**) Carvone stimulation (300 μM) of healthy platelets promoted chloride efflux. Data are shown as the mean ± SEM (*n =* 4, MQAE fluorescence). *P = 0.0134, (–) carvone versus control, by 2-tailed Student’s *t* test. (**D**) Carvone stimulation (300 μM) of healthy platelets promoted local brief calcium transients 20%–80% above baseline, which was sustained compared with 0.5 U/mL thrombin that attenuated over time (*n =* 5 human platelets, Fura-2 fluorescence). MQAE, -[ethoxycarbonylmethyl]-6-methoxy-quinoliniumbromide. Arrowhead, drug addition.

**Figure 6:**
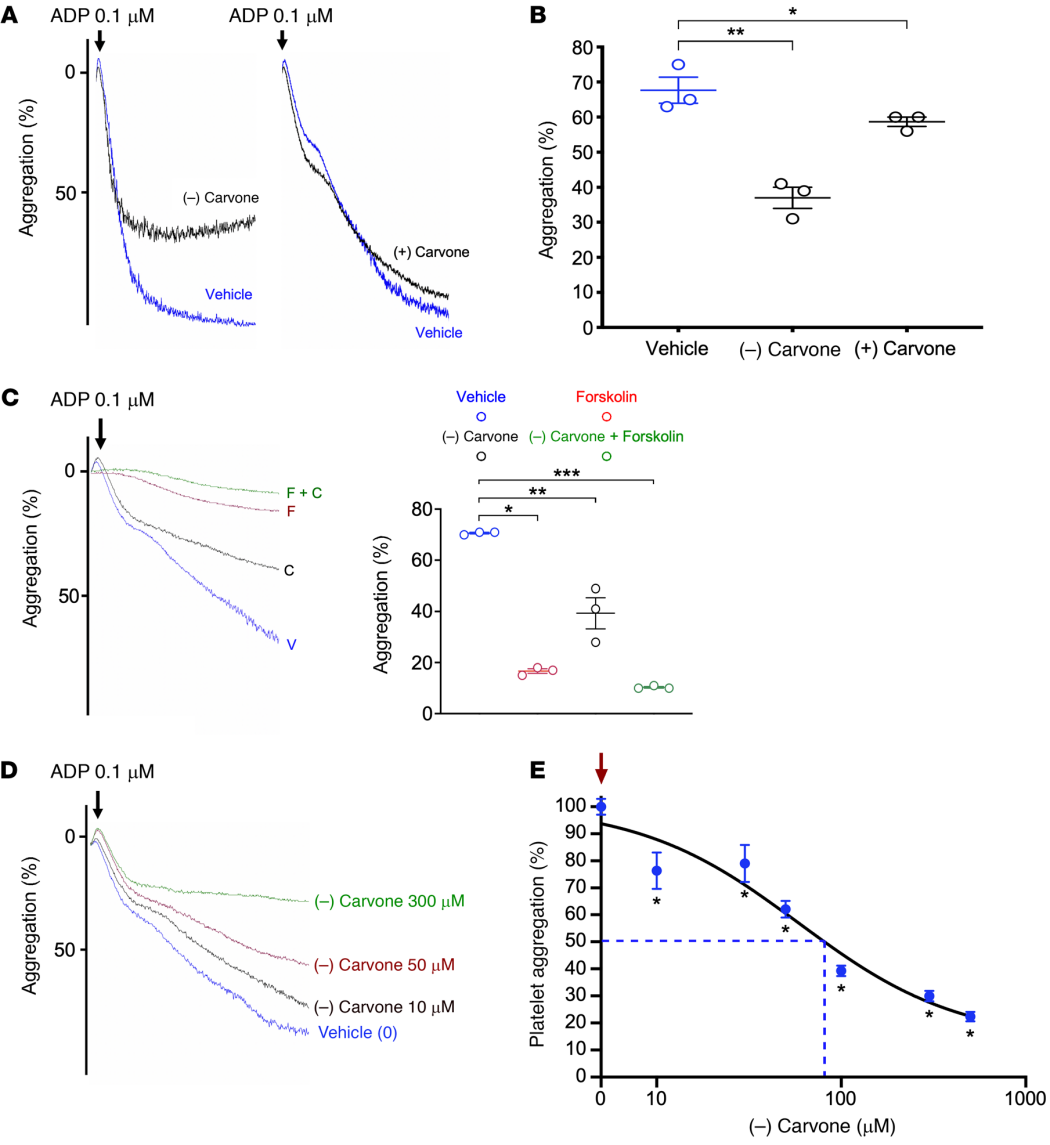
A OR2L13 agonist potently inhibits platelet aggregation. (**A**) Platelets were isolated from healthy individuals and preincubated with (–) carvone or (+) carvone (300 μM) for 30 minutes. Platelets were stimulated with ADP (0.1 μM), and light transmission aggregometry was performed to assess platelet activation. Representative tracings for each agonist are shown. (**B**) Carvone (300 μM) or forskolin (10 μM) incubation individually or together for 30 minutes followed by platelet stimulation with ADP (0.1 μM). Light transmission aggregometry was performed to assess platelet activation. Representative aggregometry tracings are shown. V, vehicle; C, (–) carvone, f, forskolin. *n =* 3. Differences between groups were assessed by 1-way ANOVA followed by Bonferroni’s correction; *P < 0.0001, **P = 0.0003, and ***P < 0.0001 versus vehicle. (**C**) Summary data from light transmission aggregometry for each agonist are presented as the mean ± SEM. *n =* 3. *P = 0.08 versus vehicle and **P = 0.0013 versus vehicle, by 1-way ANOVA followed by Bonferroni’s correction. (**D**) Platelets were isolated from healthy individuals and preincubated with (–) carvone (0–500 μM) for 30 minutes, followed by stimulation with ADP (0.1 μM). Light transmission aggregometry was performed to assess platelet activation. Representative tracings for each agonist are shown. (**E**) Summary data for each concentration of (–) carvone are presented as the mean ± SEM in the presence of ADP stimulation (0.1 μM). The downward red arrow is ADP in the presence of vehicle to which all data points were compared. *n =* 3 in each group. *P < 0.05 versus vehicle, by 1-way ANOVA followed by Bonferroni’s correction. The broken blue line indicates the log IC_50_ concentration of (–) carvone.

Olfactory receptors and downstream anoctamin proteins are generally expressed in afferent olfactory neurons (23). Olfactory receptors are GPCRs positively linked to adenylyl cyclase to increase cAMP (24), triggering anoctamin 7 as a downstream calcium-sensitive chloride channel (25). If this signal transduction pathway is conserved in platelets, OR2L13 activation should generate cAMP in platelets coincident with changes in platelet Cl^–^ and Ca^2+^ flux (schematic, Figure 5A). We indeed found (–) carvone generated cAMP in platelets and inhibited platelet aggregation compared with forskolin, which generated cAMP in a receptor-independent manner (Figure 5B). Consistent with the predicted signal transduction pathway in olfactory neurons, platelet OR2L13 activation by (–) carvone promoted platelet Cl^–^ efflux and brief Ca^2+^ transients, presumably as downstream components of platelet postreceptor signal transduction (Figure 5, C and D). Carvone exists as levo (–) and dextro (+) enantiomers. We found the (–) enantiomer of carvone to have a more potent antiplatelet effect (Figure 6, A and B) and (–) carvone blunted ADP-induced platelet aggregation dose dependently (Figure 6, C–E).

**Figure 7:**
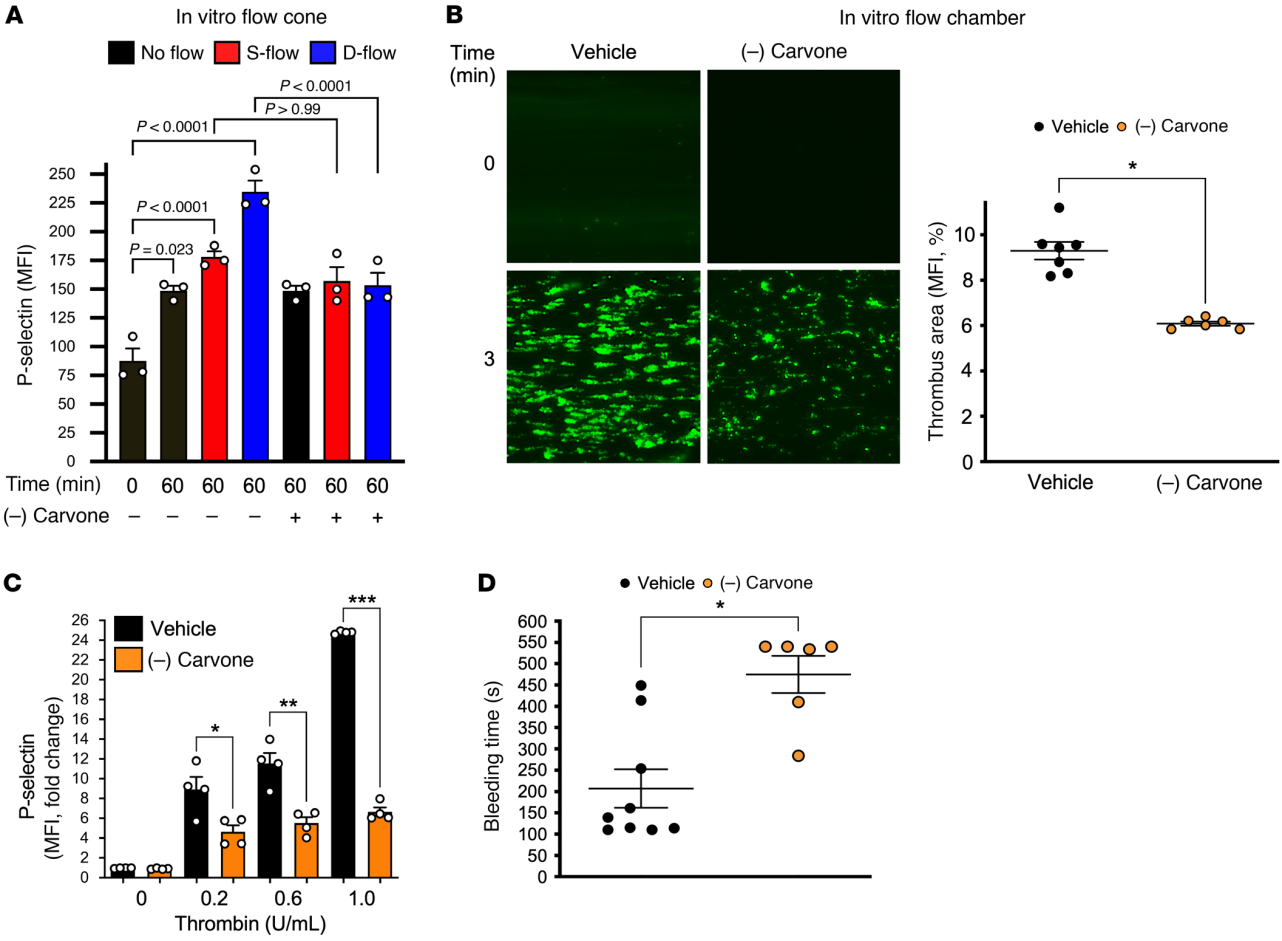
Biomechanical platelet activation is inhibited by OR2L13 agonists. (**A**) In vitro exposure of healthy platelets to steady laminar flow (S-flow) or disturbed flow (D-flow) for 60 minutes after a 30-minute pretreatment with vehicle or (–) carvone (300 μM). Platelet activation was quantified by surface P selectin expression as the MFI ± SEM. *n =* 3 in each group; 2-way ANOVA. (**B**) Calcein green–loaded healthy human blood flowing through a collagen I–coated microfluidics chamber at 40 dyn/cm^2^ and imaged by confocal microscopy for adherent thrombus (green puncta) at 3 minutes. Data are presented as the mean thrombus area of random fields ± SEM following 30 minutes of vehicle (0.25% DMSO) or (–) carvone treatment (300 μM). *n =* 6–7 in each group. Differences between groups were assessed by 2-tailed Student’s *t* test; *P < 0.0001 versus vehicle. (**C**) WT FVB/Tac mice were given 100 mg/kg/day (–) carvone i.p. for 3 days. Platelets were isolated and stimulated in the presence of thrombin. Platelet activation was quantified by FACS as the MFI ± SEM. *n =* 4. *P = 0.061, **P = 0.007, and ***P < 0.0001 versus vehicle, by 1-way ANOVA followed by Bonferroni’s correction. (**D**) Time to hemostasis in mice treated with (–) carvone following surgical amputation of tail tip in seconds ± SEM. *n =* 6–9 in each group. *P = 0.0046 versus vehicle, by Mann-Whitney *U* test.

We next evaluated biomechanical platelet activation by S-flow and D-flow. Human platelets exposed to D-flow became activated and OR2L13 localized to the membrane surface. We incubated healthy platelets with (–) carvone or control buffer before subjecting them to S-flow or D-flow, observing (–) carvone attenuated only D-flow–induced platelet activation (Figure 7A). Utilizing another model of biomechanical platelet activation, vehicle- or (–) carvone–treated human blood was passed through a collagen-coated microfluidic perfusion chamber to determine the rate of thrombus formation at high shear. Thrombosis in whole blood was attenuated by (–) carvone (Figure 7B and Supplemental Video 1, A and B).

To ascertain whether exogenous (–) carvone in mice affected platelet reactivity and thrombosis in vivo, FVB/Tac mice were administered (–) carvone at a dose of 100 mg/kg/day for 3 days by i.p. injection. Platelets were then isolated from mice and thrombin-stimulated ex vivo. Thrombin-induced platelet degranulation ex vivo was inhibited in platelets from (–) carvone–treated mice (Figure 7C). Tail bleeding times in mice treated with (–) carvone were also prolonged (Figure 7D) without affecting the circulating platelet number (Supplemental Figure 7). This effect was consistent with adequate absorption and distribution of (–) carvone to attenuate platelet activation and thrombosis in vivo.

### Platelets are mechanically activated in AAA, which directs OR2L13 to the membrane.

**Figure 8:**
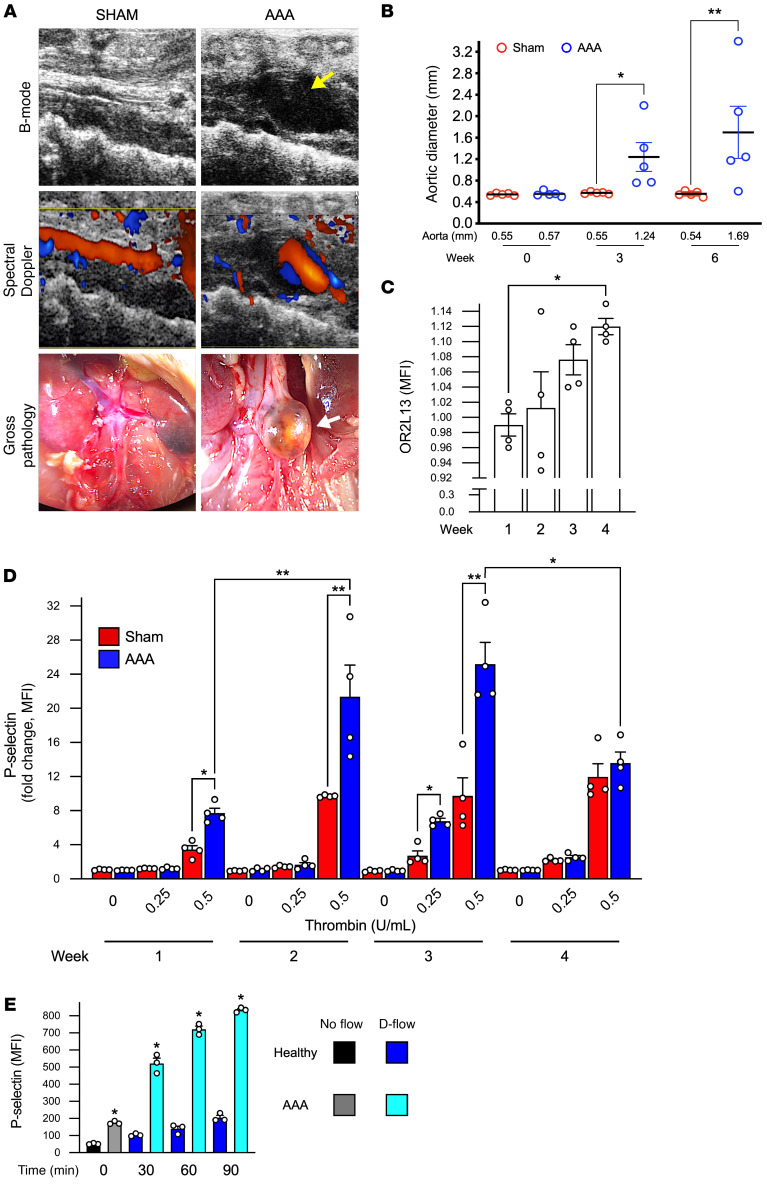
Platelets are biomechanically activated in mice and humans with AAA. (**A**) WT male C57BL/6J mice treated with topical aortic elastase or heat-inactivated elastase (sham) with BAPN in drinking water developed stable AAA with luminal thrombus (yellow arrow). Top: B-mode ultrasound, middle: color spectral Doppler interrogation of the aneurysmal region shows D-flow (Doppler [red alternating to blue]) bottom: dissecting video microscopy at the end of the protocol with marked aneurysm (white arrow) below the renal artery. Time point is 6 weeks after AAA. (**B**) Aortic diameter by ultrasound is shown as the mean ± SEM (diameter indicated below the graph). *n =* 5. *P = 0.114 and **P = 0.0046, by repeated-measures 1-way ANOVA followed by Bonferroni’s correction. (**C**) Platelet surface OR2L13 expression for translocation from baseline and after 4 weeks of AAA induction as the MFI ± SEM. *n =* 4. *P = 0.0251, by 1-way ANOVA followed by Bonferroni’s correction. (**D**) Platelet surface P selectin for platelet activation after dose-dependent thrombin stimulation in sham-operated or AAA mice in weeks 1–4 after AAA induction. *n =* 4 in each group. Differences between groups were determined by 2-way ANOVA; *P < 0.01 and **P < 0.001. (**E**) An in vitro flow-and-cone system was used to subject healthy platelets to static flow (0) or disturbed flow (D-flow) for 0–90 minutes. Platelet activation as the mean surface P selectin ± SEM, *n =* 3 in each group, by 1-way ANOVA followed by Bonferroni’s correction.

We recently reported that aspirin limits AAA growth in a large clinical population (10). Mice were injected 3 times weekly with an anti–TGF-β antibody to induce aneurysmal growth and promote luminal thrombus formation. (26). Topical elastase was applied to the infrarenal aorta of mice to degrade elastin with the lysyl oxidase inhibitor β−aminopropionitrile (BAPN) administered ad libitum in the drinking water to prevent elastin cross-linking repair (26). To investigate a mechanism for this clinical observation, we explored the function of OR2L13 in a murine model of fast-growing AAA that developed luminal thrombus, similar to human infrarenal AAA in which D-flow was observed in aneurysmal aortic regions (Supplemental Videos 2 and 3, ref. 26, and Supplemental Figures 8 and 9). We confirmed this model of AAA exhibited D-flow in the aneurysmal segment and occasional luminal thrombus formation (Figure 8A). After 6 weeks, the aortic diameter increased 300% above baseline, consistent with severe AAA. Coincident with AAA growth, enhanced agonist-mediated platelet reactivity was observed, as well as increased surface OR2L13 expression in isolated platelets. Mice with AAA demonstrated enhanced platelet reactivity in which a D-flow environment pathologically appeared compared with sham-operated mice with S-flow, reaching a plateau at 3 weeks and then dissipating at 4 weeks coincident with increasing platelet surface OR2L13 expression (Figure 8, B–D). Furthermore, platelets isolated from patients with AAA were markedly sensitized to biomechanical activation ex vivo by D-flow compared with healthy platelets in an S-flow environment (Figure 8E).

### OR2L13 agonists inhibit platelet activation and AAA growth.

**Figure 9:**
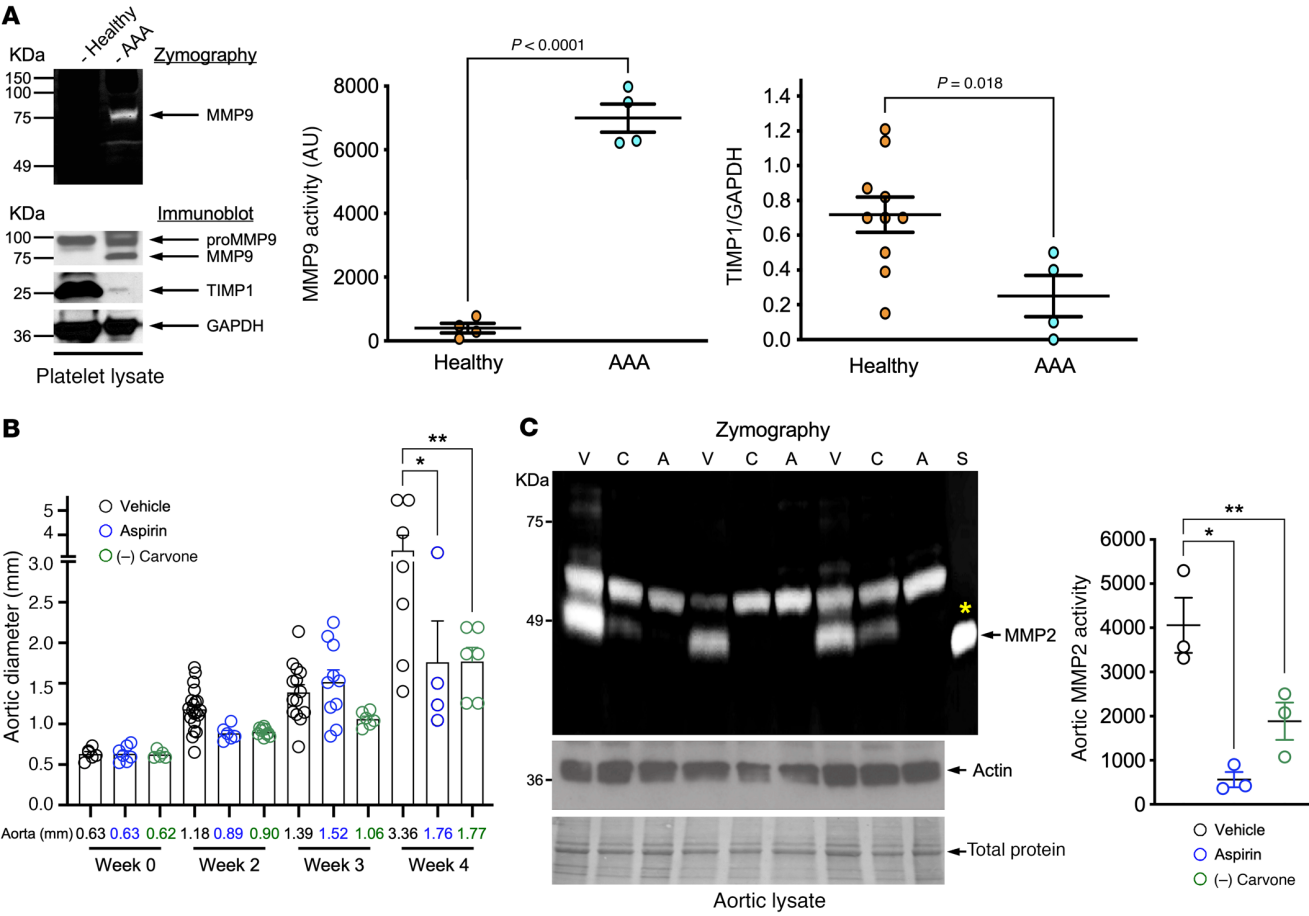
Platelet OR2L13 agonists suppress platelet reactivity and AAA growth. (**A**) Nonreducing SDS-PAGE of human platelet lysates for MMP activity was examined by in-gel zymography, and protein content was examined by immunoblotting. MMP9 content was similar, although activated MMP was enriched in platelets from patients with AAA compared with those from healthy individuals (*n =* 4–5). Data were quantified as the mean ± SEM and normalized to GAPDH (*n =* 4–10). Differences between groups were determined by 2-tailed Student’s *t* test. TIMP, tissue inhibitor of MMP. Protein size is indicated in kDa. (**B**) Aortic diameter by ultrasound following aspirin (30 mg/L, drinking water) therapy or daily i.p. injection of 100 mg/kg (–) carvone compared with vehicle starting on day 7 protected FVB/NTac mice from fast AAA growth (*n =* 4–20). *P = 0.0002 and **P < 0.0001 versus vehicle, by 1-way ANOVA followed by Bonferroni’s correction. (**C**) Aortic lysate at week 4 following AAA was assessed for MMP activity (zymography). Actin and total protein were used as loading controls. Data are representative of 9 WT mice (*n =* 3 in each group) at 4 weeks. *P = 0.012 and **P = 0.0002 versus vehicle, by 2-way ANOVA. Yellow asterisk indicates a purified and activated MMP2 standard (S). Protein size is indicated in kDa.

We previously reported that platelet MMP activity is increased in myocardial infarction (7, 8). Given that patients with AAA show less aortic growth and rupture when taking aspirin and growth and rupture are linked to MMP activity (10), we hypothesized that circulating platelets in patients with AAA may synthesize and secrete MMPs. Platelet MMP9 activity was enhanced and the tissue inhibitor of MMP9 (TIMP1) was reduced in AAA compared with healthy conditions (Figure 9A). Platelet MMP9 content was unchanged in healthy compared with AAA platelets (not shown). Aortic tissue as well as luminal thrombus from patients with infrarenal AAA compared with nonaneurysmal cadaveric aorta had increased expression and activity of MMP9. This observation suggests that platelets, which are a component of luminal thrombus, may contribute to aortic remodeling through MMPs (Supplemental Figure 10). In our murine aneurysm model (26), (–) carvone treatment attenuated AAA growth similarly to aspirin, further suggesting antiplatelet therapeutics restrict aortic growth (Figure 9B and Supplemental Figure 11). Consistent with the hypothesis that platelet-derived mediators regulate AAA growth, mice administered daily (–) carvone showed suppressed AAA growth coincident with decreased MMP2 activity in the aorta (Figure 9C). The inhibitory effect of (–) carvone on aortic growth and MMP activation was similar to — but slightly more potent than — what was observed in aspirin-treated animals and suggests a platelet-mediated effect in regulating AAA progression (Figure 9C).

**Figure 10:**
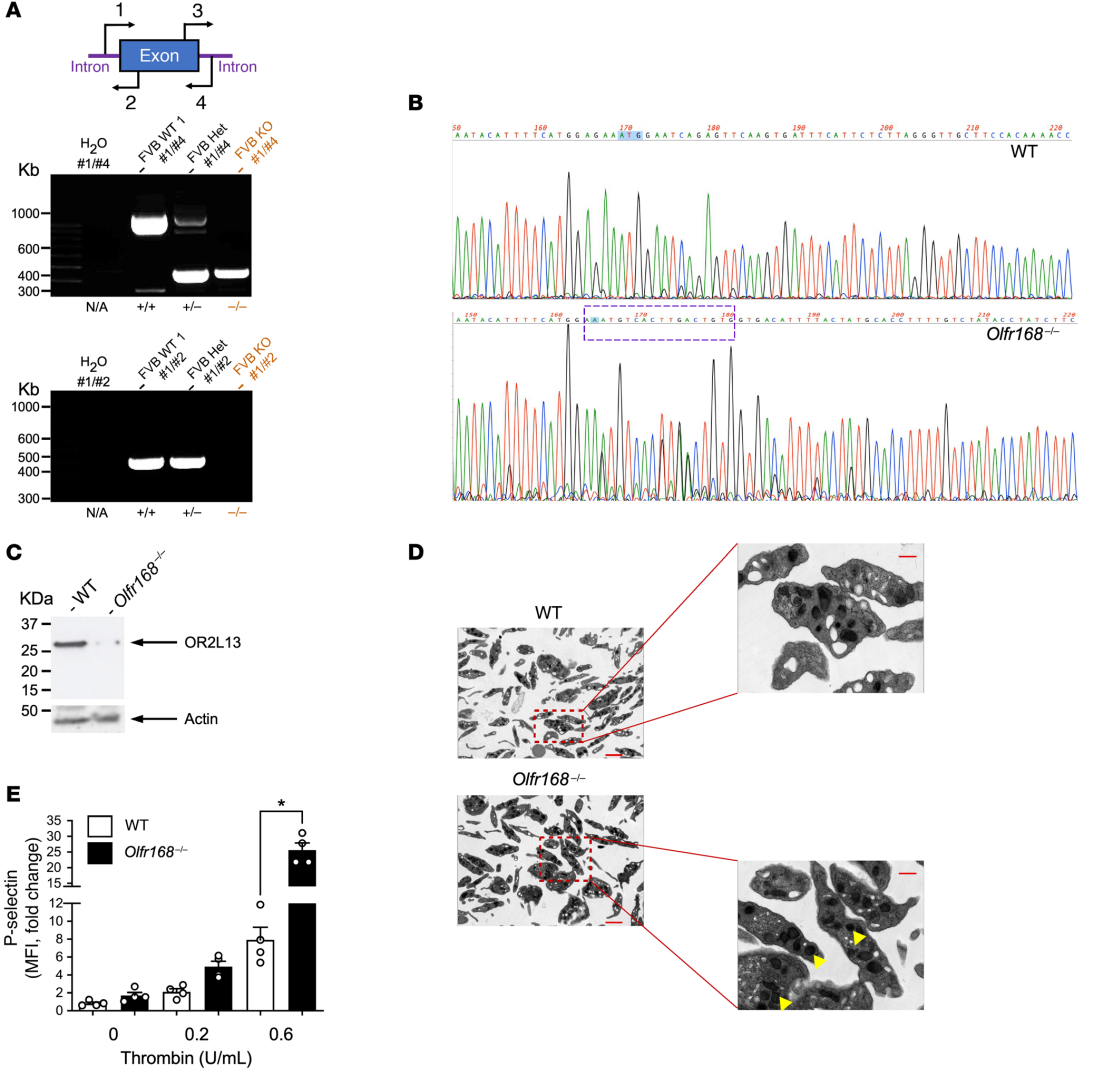
CRISPR/Cas9 disruption of the *Olfr168* locus augments platelet reactivity. (**A**) Sequencing primer design spanning the OR2L13 (FVB/Tac murine *olfr168*) intron/exon boundaries. Genotyped pups after RNP injection showed the edit (arrow) following PCR with #1/#4 primers and deletion following PCR with #1/#2 primers (right gel, last lane). (**B**) Sanger sequencing showed an edit in the upstream region of the olfr168 locus (box). Amplified product size is indicated in kb. Het, heterozygote. KO was *olfr168*^–/–^. (**C**) Immunoblotting platelet lysate for the protein product of the FVB/Tac (WT) and null (KO) murine alleles for the *olfr168* gene using an anti-OR2L13 antibody. (**D**) Electron micrographic images of individual WT and olfr168^–/–^ mouse platelets with increased thrombotic granule content apparent in olfr168^–/–^ mouse platelets (arrowheads). Scale bars: 0.5 μm (magnified images). (**E**) Isolated *olfr168*^–/–^ platelets showed increased reactivity when stimulated with thrombin ex vivo. Platelet activation is shown as the mean surface P selectin ± SEM. *n =* 4 in each group. *P < 0.0001, by 1-way ANOVA followed by Bonferroni’s correction.

**Figure 11:**
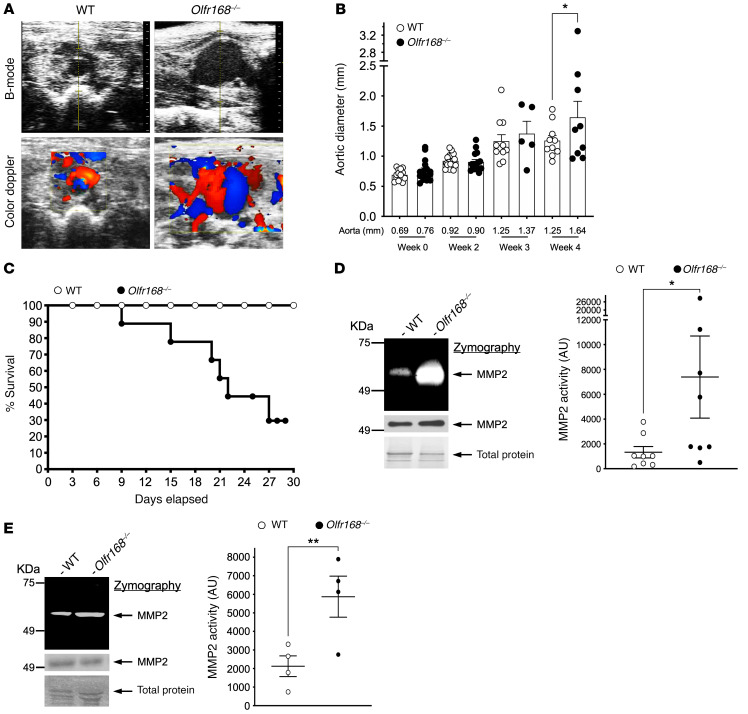
*Olfr168-*deficient mice have enhanced platelet and aortic MMP2 activation with accelerated aortic aneurysm growth and rupture. (**A**) B-mode ultrasound of infrarenal AAA at week 4 after elastase induction and color spectral Doppler showing marked D-flow in AAA. Bottom: Color spectral Doppler of aortic aneurysmal segments showing disturbed blood in *olfr168* ^–/–^ mice. (**B**) AAA growth by ultrasound for WT or olfr168^–/–^ FVB/Tac mice (*n =* 5–17). *P = 0.0037 WT versus *olfr168*^–/–^, by Kruskal-Wallis test followed by Dunn’s post test. (**C**) Kaplan-Meier survival curves for AAA rupture in WT or *olfr168*^–/–^ FVB/Tac mice (*n =* 10 in each group). Differences between groups were evaluated with the log-rank (Mantel-Cox) test; *P =* 0.042 between groups. (**D**) Aortic lysate following AAA was assessed for MMP activity (zymography) in FVB/Tac mice at 4 weeks (*n =* 8 in each group). *P = 0.028 versus Olfr168^–/–^, by Mann-Whitney *U* test. MMP2 and total protein (stain) were used as loading controls. (**E**) Platelet lysate following AAA was assessed for MMP2 activity (zymography) in FVB/Tac mice at 4 weeks (*n =* 4 in each group). *P = 0.023 versus Olfr168^–/–^, by 2-tailed Student’s *t* test.

We examined platelets to determine the expression of the murine OR2L13 homologue *olfr168* in various strains of mice and found the FVB/Tac strain expressed OR2L13 at the greatest level compared with C57BL6/J mice that expressed little OR2L13 (Supplemental Figure 12, A–C). Using CRISPR/Cas9, we made an upstream edit in a unique sequence within the open reading frame of murine *olfr168* resulting in gene deletion (Figure 10, A and B). Immunoblotting-washed murine platelet lysate confirmed the absence of OR2L13 (*olfr168*^–/–^) (Figure 10C). Electron microscopic visualization of platelets showed enhanced granule content in *olfr168*^–/–^ mice coincident with enhanced platelet reactivity ex vivo compared with WT littermates (Figure 10, D and E). This further suggests *olfr168* may be an endogenous negative regulator of platelet function. Comparing WT with *olfr168*^–/–^ mice in the AAA model, the magnitude of D-flow observed in aneurysmal arterial segments, the rate of aortic growth, and incidence of aortic rupture all increased in mice with *olfr168* deficiency (Figure 11, A–C). Finally, *olfr168*^–/–^ mice had augmented aortic MMP2 activity compared with WT mice (Figure 11D) and isolated platelets from either WT mice treated with the *olfr168* agonist (–) carvone or from *olfr168*^–/–^ mice had decreased and increased MMP2 activity compared with control mice, respectively (Figure 11E and Supplemental Figure 13). Deficiency of *olfr168* did not affect platelet count, WBC count, or hemoglobin concentration at baseline or after AAA induction (Supplemental Figure 14). Together, these data imply that activated platelet OR2L13 is a protective mechanism to prevent excessive platelet reactivity in AAA and simultaneously silences platelet MMP2 activity. This may in part provide a mechanistic explanation for antiplatelet agents restricting AAA growth and rupture (4, 10).

## Discussion

To the best of our knowledge, this is the first study to show a mechanistic link between platelet reactivity and AAA progression in vivo, identifying functional olfactory receptors as signal transduction modules in platelets to serve as druggable targets. This discovery was made in aortic aneurysmal disease where platelet OR2L13 appears to be upregulated in response to biomechanical activation and, specifically, by external D-flow exposure. Olfactory receptors are GPCRs that activate adenylyl cyclase to hydrolyze cAMP from ATP, a well-known second messenger that suppresses platelet reactivity (27). We identified the terpene (–) carvone, an active ingredient in spearmint, as a potent platelet OR2L13 agonist that generates cAMP endogenously in platelets. The same OR2L13 agonist suppresses thrombosis and platelet reactivity both ex vivo and in vivo. OR2L13 activators are therefore foundational to a new class of antiplatelet agents for thrombotic diseases.

In aneurysmal regions of the mouse and human aorta, D-flow and luminal thrombus are common observations and may accelerate AAA growth (28–30). We showed a similar fingerprint of MMP9 activity in platelets, aortic thrombus, and aorta in humans with advanced AAA. We found that patients with AAA had markedly reduced platelet TIMP1 expression. TIMP1 is an inhibitor of MMP9 (31). Much like OR2L13 in this study, TIMP1 is stored in platelet alpha granules and secreted upon platelet activation (32). Platelet proteomic analysis previously demonstrated that TIMP1 expression inside platelets dramatically changes with aspirin pretreatment (33).

In a murine model of AAA, aspirin or the platelet OR2L13 agonist (–) carvone treatment suppressed platelet and aortic MMP2 activity, which restricted AAA growth. Similarly, OR2L13-deficient mice had increased platelet granular content and reactivity and augmented platelet and aortic MMP activity — observations that coincide with enhanced AAA growth and early rupture. Global *olfr168*^–/–^ mice were used as proof of principle that OR2L13 protects against platelet activation, platelet and aortic MMP activation, and aortic rupture in AAA, but an intrinsic limitation is a lack of availability of mice with platelet-specific *olfr168* deletion. Although some mice with AAA form thrombi in the aneurysmal aorta resembling the human condition, we did not see this in every case, which makes this connection challenging to incorporate into the final mechanistic explanation. Nonetheless, utilizing human AAA aortic tissue and a murine model, these findings suggest platelet-derived MMPs may contribute to the pathogenesis of AAA.

Olfactory receptor signaling in nonolfactory tissue is a relatively new discovery but appears to regulate physiological processes, including blood pressure, fat metabolism, and airway hyperresponsiveness (34–36). Hereditary anosmia (the inability to smell using functional olfactory receptors) coexists in patients with dysfunctional platelet activity — an observation made many years ago that was never explored (37). In addition, during the SARS-CoV-2 pandemic from 2019 to 2021, anosmia, presumably through inactivation of olfactory receptors, was observed as a common initial symptom of infection, and platelet activation as well as thrombosis are clear sequelae of this disease (38–40). These clinical observations raise the possibility that platelet olfactory receptors are mechanistic explanations for dysregulated platelet function in certain diseases. Of the several hundred olfactory receptors in the human genome, we determined only a few were expressed in the platelet precursor megakaryocyte, and even fewer at the protein level in mature adult platelets. Healthy platelets express reasonable quantities of OR2L13, OR2W2, and OR2B6. OR2L13 was the only platelet olfactory receptor to change expression in AAA and so became a natural target for our investigation.

We showed that OR2L13 colocalized with P selectin in platelets, suggesting alpha granule mobilization may be responsible for trafficking OR2L13 to the plasma membrane when platelets are activated by D-flow and further emphasizing that OR2L13 has a protective role, attempting to attenuate activation of platelets persistently exposed to mechanical stimuli. We demonstrated using an OR2L13 ligand screen in vitro that (–) carvone is an OR2L13 agonist capable of increasing platelet cAMP, which inhibits platelets, thrombosis, and AAA progression in vivo. The fact that OR2L13 activation increases cAMP production, chloride efflux, and transient calcium mobilization in platelets, similar to its native expression in olfactory afferent neurons, suggests a conserved signal transduction pathway (41–43). Over a decade ago, our group demonstrated platelets possess other functional signal transduction pathways common to neurons, including the NMDA receptor and the alpha-amino-3-hydroxy-5-methyl-4-isoxazolepropionic acid (AMPA) receptor (44). Recently, muscarinic acetylcholine receptors were found to regulate platelet reactivity and thrombosis (45). These previous studies and the current observations suggest that circulating platelets resemble neuronal synapses.

In patients with AAA, we demonstrated that P2Y_12_ receptor activation, which decreased platelet cAMP, was slightly inhibited, unlike PAR1 and the platelet thromboxane receptors, which showed enhanced activity in AAA. Patients with AAA have increased platelet OR2L13 expression, a receptor pathway that increases cAMP, suggesting development of a protective mechanism involving enhanced adenylyl cyclase activity and cAMP production in circulating platelets of patients with AAA. Several platelet surface GPCRs positively couple to adenylyl cyclase to generate cAMP (46). OR2L13 agonists could be alternative antiplatelet agents for patients with cardiovascular disease in whom P2Y_12_ receptor antagonists are ineffective (47, 48). In a recent clinical trial in patients with small infrarenal aortic aneurysms, a P2Y_12_ receptor antagonist did not impair aneurysmal growth when administered in a randomized, blinded manner (14). The P2Y_12_ receptor, therefore, may play a smaller role in platelet-mediated AAA growth, as suggested by our data. Alternatively, medical intervention for small aortic aneurysms or the time of treatment during AAA development in the prior report may generate an insufficient magnitude of D-flow to activate platelets.

We discovered that platelets from patients with AAA were biomechanically activated in spite of aspirin therapy but can be suppressed by the OR2L13 agonist (–) carvone. Parenteral administration of (–) carvone in mice inhibited the increase in platelet reactivity and suppressed AAA growth in vivo. Detailed pharmacokinetic and pharmacodynamic studies will be required to assess the bioavailability of carvone, which is found in spearmint extract and therefore could be administered orally. Since olfactory receptor agonists are often volatile odors, the possibility of engineering an antiplatelet agent for aerosolized delivery exists.

In summary, this investigation identified functional platelet olfactory receptors and showed that surface OR2L13 stimulation suppressed platelet reactivity in humans and in mice. When platelet OR2L13 was activated during AAA development by an exogenous, activating ligand, the biological consequence was suppression of platelet and aortic MMP activity, limiting aortic aneurysm progression. Conversely, OR2L13 deficiency augmented platelet and aortic MMP activity, promoting aneurysmal growth and aortic rupture. Future work will be directed toward understanding bidirectional communication between the aorta and platelets to better understand additional mechanisms by which platelets contribute to aortic remodeling.

## Methods

For detailed information on common and routine experimental procedures, refer to the Supplemental Methods.

### Odorant screen.

The control and RTP1s/OR2L13 reporter cells were used to perform odorant screening. Briefly, 10,000 reporter cells (control or RTP1s/OR2L13 expressing) were plated per well of a 384-well cell culture plate in 10 μL complete growth media without phenol red. Immediately after cell plating, 10 L of 0 to 500 μM as an alpha screen odorant mix was added to each well in duplicate and gently vortexed. The screen was then repeated with odorant molecules individually in a dose-dependent manner. Forskolin (1 μM) and DMSO were added to 1 column of every assay plate to serve as a control. After the odorant mix addition, compounds from the Spectrum Collection (MS Discovery) were added to each well at approximately 1 μM concentration and incubated overnight at 37°C in 5% CO_2_. The next day, 20 μL of One-Step Luciferase assay system (BPS Bioscience) was added to each plate, and luciferase activity was read on a Gen5 plate reader. The ratio or OR2L13 cell line/vector cell line was used to indicate a positive response by way of a cAMP response element reporter readout ratio of more than 1.0; then a dose-response curve was constructed for lead compounds.

### Ex vivo exposure of platelets to mechanical stress.

Healthy human blood was loaded with the fluorophore calcein green at room temperature (10 μM, pH 7.4 for 20 minutes), and incubated with odorant ligands for 30 minutes. Using a Cellix Microfluidics System, blood was perfused over a collagen I–precoated Vena8 Fluoro+ biochip (15 μL at 150 μg/mL, humidified box overnight at 4°C, then washed with PBS) at 40 dyn/cm^2^ using a Mirus Nanopump. Image collection was conducted using an HC Plan Apo 20X/0.7NA lens mounted to a Leica DMI6000 inverted microscope and a dark chamber and a Hamamatsu ImagEM cooled, charge-coupled device camera. Platelets loaded with calcein green were used to determine thrombus size as percentage thrombus area at the end of 3 minutes using Image Pro plus software (Media Cybernetics). Platelet exposure to S-flow (smooth surface) and D-flow (radial grooves) in vitro was made possible using a flow cone and plate system custom-manufactured by the University of Rochester department of engineering as described previously (19). The cone was rotated to provide S-flow shear at 15 dyn/cm^2^. D-flow shear cannot be accurately determined, though the same rotational speed was used with S-flow and D-flow always run alongside no-flow (static) conditions for internal consistency. After exposure to S-flow or D-flow, platelets were imaged by confocal microscopy, surface marker proteins were determined quantitatively by flow cytometry, and platelet proteins were assessed by Western blotting.

### Experimental animals.

Mouse colony: 8-week-old male WT C57/BL6 (The Jackson Laboratory) and FVB/NTac (Taconic Biosciences) mice were used in this study given that AAA is a disease affecting mostly males. The murine ortholog OR2L13 gene (*olfr168*) deletion mice were created in the University of Rochester functional genomic core using a CRISPR/Cas9 editing strategy with injection of the following guide RNA strands (Synthego Corporation): CAAGTGATTTCATTCTCTTA and GGGCCATGACAAGAGTCCTT. Genotyping using primers spanning the predicted gene edit location were utilized and pups were further confirmed by Western blotting of isolated platelets using an OR2L13 antibody.

### AAA surgery.

We adapted the method demonstrated by Hu et al. to induce AAA with intraluminal thrombus (ILT), which is as close to the human phenotype of infrarenal AAA as possible (26). The experimental elements for AAA induction and intervention are shown in pictorial format (Supplemental Figure 9). BAPN at 2 g/L was given in the mouse drinking water 2 days before the surgical procedure to inhibit lysyl oxidase, which would otherwise repair the damage to the aorta caused by topical elastase. To augment aneurysm growth and induce ILT as described (26), anti−TGF-β blocking antibody (250 μg/mouse) was i.p. injected 3 times weekly starting the day of surgery. Each mouse (20−30 g in weight) was given 1 mL s.c. physiologic saline before surgical incision to account for insensible volume losses with gut externalization. Surgical procedures were conducted on a surgical heating pad at 37°C. Mice were anesthetized with isoflurane anesthesia (3% by nosecone) using oxygen as the carrier. Buprenorphine (0.05 mg/kg) was given 30 minutes prior to skin incision. The area of incision was cleaned with 70% ethanol and betadine 3 times, and then infiltrated gently with local anesthesia (bupivacaine 5 mg/kg, s.c., immediately prior to incision). A midline abdominal incision was made through the skin. The rectus sheath was divided at the linea alba. The intestines were exteriorized with a Q-tip to the left of the mouse onto gauze soaked with saline above and below to prevent drying. Whatman paper 6 mm × 9 mm slices were placed on top of the aorta below the bifurcation of the renal arteries. A volume of 10 μL porcine pancreatic elastase was dropped onto Whatman paper, which was left in place for 5 minutes. For sham surgical animals, the procedure was identical, but heat-inactivated elastase was used (55°C, 10 minutes). Excess elastase was removed by adding 0.5 mL physiologic saline to the abdominal cavity followed by an aspiration step. The mesentery was repositioned in the abdominal cavity. Next, 6-0 Vicryl interrupted sutures were used to close the peritoneum, and 6−0 nylon was used to close the skin using interrupted sutures. During recovery, a heated pad was placed under the mouse cage on one half only to assist in comfort while allowing the mouse the opportunity to move to a cooler area if desired. Hyperalgesia was assessed immediately after the procedure, and then twice daily for 48 hours, and maintenance dose buprenorphine (0.05 mg/kg) was given 8 hours after surgery and then maintained at this dose for 48 hours after surgery. For the interventional study, FVB/Tac mice were injected with vehicle (DMSO) or (–) carvone starting on day 7 at the dose of 100 mg/kg/day i.p. or given vehicle (water) or aspirin 30 mg/L ad libitum in drinking water starting on day 7. Blood draws were taken at time intervals no less than 1 week by the retro-orbital route into heparinized Tyrode’s solution as we described previously (8, 20).

### Ultrasonography.

Sonographic interrogation using pulsed-wave Doppler allowed identification of the aorta. Color flow Doppler interrogation was utilized to determine the presence of D-flow in aneurysmal segments and S-flow in the aorta of sham-operated animals. After carefully scanning the aorta of mice under general isoflurane anesthesia for the widest section of the aorta in the short axis, color Doppler was used to identify the aorta and distinguish it from the vena cava. Pulsed-wave Doppler interrogated the vessel to ensure the signal was arterial, and then M-mode was used through the widest section of the short axis of the aorta for enhanced temporal resolution. Aortic dimension at the maximum lumen diameter were taken at each time point as we described previously (4). The aortic edge-to-edge intima was measured using the Vevo2100 echocardiography system (VisualSonics).

### Statistics.

Data are presented as the mean ± SEM unless otherwise stated. Normality and equal variance were first evaluated by the Shapiro-Wilk test. For normally distributed data between 2 comparative groups, a 2-tailed Student’s *t* test was used. For nonparametric data, the Mann-Whitney *U* test was used. For Gaussian-distributed data in 3 or more groups, 1-way ANOVA followed by Bonferroni’s multiple-comparison test was used, otherwise the Kruskal-Wallis test followed by Dunn’s post test was used. Significance was accepted as a *P* value of less than 0.05. Analyses were conducted using GraphPad Prism 7 (GraphPad Software).

### Study approval.

This study complies with the Declaration of Helsinki and was approved by the University of Rochester for the analysis of platelets from healthy individuals and from patients with AAA as well as blood biomarkers and aortic tissue from recently deceased individuals from nonvascular causes and from patients undergoing open aortic reconstruction. The study participants provided written informed consent. Cleveland Clinic IRB approval was obtained for the isolation of platelets from patients with AAA. All animal protocols were approved by the University Committee on Animal Resources at the University of Rochester and Cleveland Clinic IACUC.

## Author contributions

SJC, APO, CJL, S Shim, VPJ, S Stauffer, AE, SPL, and CNM conceived and designed the study. CNM, SJC, AM, MG, RB, PO, and SKT were responsible for animal care. SJC, S Shim, DM, SPL, AD, MTR, AE, and RB were responsible for phlebotomy, clinical data management, and regulation. SJC, MG, AA, AM, RB, KK, MTR, JMA, DIY, and LEW conducted laboratory testing. LEW, KK, and DM contributed to the data processing. SJC and CNM supervised all aspects of the study. CNM and SJC contributed to initial data interpretation and wrote the manuscript. All authors contributed to final data interpretation and critical revision of the manuscript and approved the final version of the manuscript.

## Supplementary Material

Supplemental data

Supplemental table 1

Supplemental video 1

Supplemental video 2

Supplemental video 3

Supplemental video 4

## Version 1 03/24/2022

In-Press Preview

## Version 2 05/02/2022

Electronic publication

## References

[1] Howard DP (2013). Population-based study of incidence and outcome of acute aortic dissection and premorbid risk factor control: 10-year results from the Oxford Vascular Study. Circulation.

[2] Sakalihasan N (1996). Activated forms of MMP2 and MMP9 in abdominal aortic aneurysms. J Vasc Surg.

[3] Alvarez Marcos F (2017). Effect of antiplatelet therapy on aneurysmal sac expansion associated with type II endoleaks after endovascular aneurysm repair. J Vasc Surg.

[4] Owens AP (2015). Platelet inhibitors reduce rupture in a mouse model of established abdominal aortic aneurysm. Arterioscler Thromb Vasc Biol.

[5] Robless PA (2003). Increased platelet aggregation and activation in peripheral arterial disease. Eur J Vasc Endovasc Surg.

[6] Hansen KB (2015). Mechanical platelet activation potential in abdominal aortic aneurysms. J Biomech Eng.

[7] Faraday N (1997). Gender differences in platelet GPIIb-IIIa activation. Thromb Haemost.

[8] Cameron SJ (2015). Platelet extracellular regulated protein kinase 5 is a redox switch and triggers maladaptive platelet responses and myocardial infarct expansion. Circulation.

[9] Nishiguchi T (2016). Local matrix metalloproteinase 9 level determines early clinical presentation of st-segment-elevation myocardial infarction. Arterioscler Thromb Vasc Biol.

[10] Elbadawi A (2020). Antiplatelet medications protect against aortic dissection and rupture in patients with abdominal aortic aneurysms. J Am Coll Cardiol.

[11] Chen Y (2019). An integrin α_IIb_β_3_ intermediate affinity state mediates biomechanical platelet aggregation. Nat Mater.

[12] Bhagavan D (2018). Strongly coupled morphological features of aortic aneurysms drive intraluminal thrombus. Sci Rep.

[13] Haller SJ (2018). Intraluminal thrombus is associated with early rupture of abdominal aortic aneurysm. J Vasc Surg.

[14] Wanhainen A (2020). The effect of ticagrelor on growth of small abdominal aortic aneurysms-a randomized controlled trial. Cardiovasc Res.

[15] Malnic B (2004). The human olfactory receptor gene family. Proc Natl Acad Sci U S A.

[16] Ito Y (2018). Turbulence activates platelet biogenesis to enable clinical scale ex vivo production. Cell.

[17] Soo Kim B (2021). Sex-specific platelet activation through protease-activated receptors reverses in myocardial infarction. Arterioscler Thromb Vasc Biol.

[18] Godwin MD (2022). Sex-dependent effect of platelet nitric oxide: production and platelet reactivity in healthy individuals. JACC Basic Transl Sci.

[19] Heo KS (2011). PKCζ mediates disturbed flow-induced endothelial apoptosis via p53 SUMOylation. J Cell Biol.

[20] Cameron SJ (2018). Hypoxia and ischemia promote a maladaptive platelet phenotype. Arterioscler Thromb Vasc Biol.

[21] Weyrich AS (2009). Protein synthesis by platelets: historical and new perspectives. J Thromb Haemost.

[22] Wu L (2012). Receptor-transporting protein 1 short (RTP1S) mediates translocation and activation of odorant receptors by acting through multiple steps. J Biol Chem.

[23] Zak JD (2018). Calcium-activated chloride channels clamp odor-evoked spike activity in olfactory receptor neurons. Sci Rep.

[24] Nakashima N (2020). Olfactory marker protein directly buffers cAMP to avoid depolarization-induced silencing of olfactory receptor neurons. Nat Commun.

[25] Duran C, Hartzell HC (2011). Physiological roles and diseases of Tmem16/Anoctamin proteins: are they all chloride channels?. Acta Pharmacol Sin.

[26] Lareyre F (2017). TGFβ (transforming growth factor-β) blockade induces a human-like disease in a nondissecting mouse model of abdominal aortic aneurysm. Arterioscler Thromb Vasc Biol.

[27] Hollopeter G (2001). Identification of the platelet ADP receptor targeted by antithrombotic drugs. Nature.

[28] Ding Y (2021). Factor Xa inhibitor rivaroxaban suppresses experimental abdominal aortic aneurysm progression via attenuating aortic inflammation. Vascul Pharmacol.

[29] Cameron SJ (2018). Antithrombotic therapy in abdominal aortic aneurysm: beneficial or detrimental?. Blood.

[30] Lindquist Liljeqvist M (2020). Tunica-specific transcriptome of abdominal aortic aneurysm and the effect of intraluminal thrombus, smoking, and diameter growth rate. Arterioscler Thromb Vasc Biol.

[31] Visse R, Nagase H (2003). Matrix metalloproteinases and tissue inhibitors of metalloproteinases: structure, function, and biochemistry. Circ Res.

[32] Villeneuve J (2009). Tissue inhibitors of matrix metalloproteinases in platelets and megakaryocytes: a novel organization for these secreted proteins. Exp Hematol.

[33] Shah P (2017). Platelet glycoproteins associated with aspirin-treatment upon platelet activation. Proteomics.

[34] Pluznick JL (2013). Olfactory receptor responding to gut microbiota-derived signals plays a role in renin secretion and blood pressure regulation. Proc Natl Acad Sci U S A.

[35] Wu C (2017). Olfactory receptor 544 reduces adiposity by steering fuel preference toward fats. J Clin Invest.

[36] Huang J (2020). The odorant receptor OR2W3 on airway smooth muscle evokes bronchodilation via a cooperative chemosensory tradeoff between TMEM16A and CFTR. Proc Natl Acad Sci U S A.

[37] Pawlowitzki IH (1986). Abnormal platelet function in Kallmann syndrome. Lancet.

[38] Hottz ED (2020). Platelet activation and platelet-monocyte aggregate formation trigger tissue factor expression in patients with severe COVID-19. Blood.

[39] Manne BK (2020). Platelet gene expression and function in patients with COVID-19. Blood.

[40] Lechien JR (2021). Prevalence and 6-month recovery of olfactory dysfunction: a multicentre study of 1363 COVID-19 patients. J Intern Med.

[41] Wright RH (1966). Odour and molecular vibration. Nature.

[42] Wright RH (1975). Odor and molecular vibration: response to nitrobenzene-d5 of honey bees (Apis mellifera L.) conditioned with nitrobenzene. Experientia.

[43] Maniati K (2017). Vibrational detection of odorant functional groups by *Drosophila melanogaster*. eNeuro.

[44] Morrell CN (2008). Glutamate mediates platelet activation through the AMPA receptor. J Exp Med.

[45] Bennett JA (2019). Acetylcholine inhibits platelet activation. J Pharmacol Exp Ther.

[46] Noe L (2010). Regulators of platelet cAMP levels: clinical and therapeutic implications. Curr Med Chem.

[47] Correll M (2013). Mutational analysis clopidogrel resistance and platelet function in patients scheduled for coronary artery bypass grafting. Genomics.

[48] Strisciuglio T (2018). Impact of genetic polymorphisms on platelet function and response to anti platelet drugs. Cardiovasc Diagn Ther.

