# Supplementary material for: Platelet olfactory receptor activation limits platelet reactivity and growth of aortic aneurysms
Source: J Clin Invest. 2022 May 2;132(9):e152373. doi: 10.1172/JCI152373 (PMC9057618; doi:10.1172/JCI152373)
Supplement: Supplemental data [file jci-132-152373-s244.pdf]

A Platelet Olfactory Receptor is a Therapeutic Target for Regulating Abdominal Aortic Aneurysm Growth

Craig N. Morrell[1], Doran Mix[2†], Anu Aggarwal[3†], Rohan Bhandari[3†], Matthew Godwin[3]

A. Philip Owens III[4], Sean P. Lyden[5], Adam Doyle[2], Krystin Krauel[6,7,8],

Matthew T. Rondina[6,7], Sharon Shim[3], Amy Mohan[1], Charles J. Lowenstein[9],

Shaun Stauffer[10], Vara Prasad Josyula[10], Sara K. Ture[1], David I. Yule[11],

Larry Wagner III[11], John M. Ashton[12], Ayman Elbadawi[13], and Scott J. Cameron [3,14,15,16] *

Running Title: Bidirectional platelet-to-aorta communication

Affiliations:
[1] Aab Cardiovascular Research Institute, Department of Medicine, Microbiology and Immunology, and Laboratory Medicine, University of Rochester School of Medicine, Rochester, New York
[2]Department of Surgery, Division of Vascular Surgery, University of Rochester School of Medicine
[3]Department of Cardiovascular and Metabolic Sciences, Lerner Research Institute, Cleveland, Ohio
[4] Division of Cardiovascular Health and Disease, Department of Internal Medicine, The University of Cincinnati College of Medicine, Cincinnati, Ohio.
[5] Heart, Vascular and Thoracic Institute, Dept of Vascular Surgery, Cleveland, Ohio
[6] Departments of Internal Medicine and Pathology, and the Molecular Medicine Program at the University of Utah Health Sciences Center, Eccles Institute of Human Genetics, Salt Lake City, Utah; & the Department of Internal Medicine and GRECC at the George E. Wahlen VAMC, Salt Lake City, Utah
[7] Molecular Medicine Program, University of Utah, Salt Lake City, Utah
[8] Department of Cardiology and Angiology I, Heart Center, University of Freiburg, Freiburg, Germany
[9] Department of Medicine, Division of Cardiology, Johns Hopkins University School of Medicine, Baltimore, Maryland.
[10] Cleveland Clinic Center for Therapeutics Discovery, Lerner Research Institute Cleveland, Ohio
[11] Department of Pharmacology and Physiology, University of Rochester School of Medicine Rochester, New York
[12]Department of Department of Biomedical Genetics, University of Rochester School of Medicine, Rochester, New York
[13]Department of Cardiovascular Medicine, University of Texas Medical Branch, Galveston, Texas
[14] Heart Vascular and Thoracic Institute, Department of Cardiovascular Medicine, Section of Vascular Medicine, Cleveland Clinic Foundation, Cleveland, Ohio
[15] Case Western Reserve University Lerner College of Medicine, Cleveland, Ohio
[16]Department of Hematology, Taussig Cancer Center, Cleveland, Ohio

Supplemental Methods
Table of antibodies and key reagents

Antibody	Clone	Isotype	References
Anti-MMP9		Human	Cell Signaling 3852S
Anti-MMP2		Human	Cell signaling 4022
Anti-OR2L13		Human	ThermoFisher Scientific PA5-48154
Anti-OR2L13		Murine	EpiGenek A66350
Anti-OR2L13-FITC		Human IgG	EpiGentek A66352
Anti-OR2W3		Human IgG	MyBioSource MBS9606652
Anti-OR2B6		Human IgG	MyBioSource MBS8513522
Anti-Anoctamin 7		Human IgG	Abcam ab196675
Anti-TIMP1		Human IgG	Abcam Ab38978
Anti-CD62P-PE	AK-4	Mouse IgG1	Invitrogen 12-0628-42
Anti-P-selectin-PE	AK-4	Mouse IgG1	Invitrogen 12-0628-42
Anti-Actin		Mouse IgG1	BD 612656
Anti-Tubulin		Mouse IgG1	Cell Signaling 3873S
Anti-GAPDH		Rabbit IgG	Cell Signaling 5174S
BAPN			Sigma A3134
Anti-TGF-β			BioXCell BE0057
Porcine Elastase			Sigma E1250-50MG
Thrombin			CaymanChemical 13188
TRAP-6			Tocris 3497

U46619			Tocris 1932
ADP			Tocris 1624

Microscopy

Glass coverslips were coated with 200 μg/mL human fibrinogen in PBS followed by aspiration and drying in a sterile hood for 30 minutes. Washed platelets were isolated and resuspended in 1 mL fresh Tyrode's solution. Washed platelets were added to the coverslip and placed in an incubator at 37 °C for 30 minutes. Excess (non-adherent) platelets were washed with fresh Tyrode's solution, followed by the addition of 2% formalin fixation for 20 minutes room temperature, then washed with Tyrode's solution. Conjugated antibody or Rhodamine-Phalloidin (3 μL) was added in PBS and placed in the dark at room temperature for 60 minutes. Excess antibody was washed with fresh Tyrode's solution, and a drop of Diamond mount was added before imaging. The surface area of platelets (μm^2) was calculated using a calibrated signal from random fields of view using an Olympus IX70 Fluorescence Microscope and a 60x oil-immersion lens.

OR2L13 cloning strategy and ligand screen

Cell culture: Stimulation of G*olf* and adenylyl cyclase was by odorant ligands were determined by cyclic AMP (cAMP) production using the cAMP/PKA Signaling Pathway CRE/CREB Reporter (Luciferase). This HEK203 cell line uses the cAMP Response Element (CRE) to drive a luciferase reporter when cAMP is made which activates PKA which, in turn, phosphorylates CRE binding protein (CREB) and promotes binding to CRE. HEK293 cells have the firefly luciferase gene under the control of multimerized cAMP response element (CRE) stably

integrated. These cells were grown in DMEM with Gultamax (Giboc cat. # 10569-010) supplemented with hygromycin B selection.

Human RTP1s and OR2L13 Cloning: Human receptor transport protein 1 subunit (RTP1s) was a kind gift from Dr. Pluznik. The cDNA for human OR2L13 in frame with hemagglutinin or green fluorescent protein (GFP) were commercially available (Sinobiological, Wayne, PA) and confirmed by Sanger sequencing. OR2L13 was cloned from pCMV3-C-GFPSpark or pCMV-HA-ORF plasmids and ligated into pLVX-IRES-mCherry or pLVX-IRES-ZsGreen (Takara, Mountain View, CA). Briefly, donor plasmids were digested with KpnI at 37ºC for 1 hour followed digest purification using Qiagen PCR Purification kit (Qiagen, Hilden, Germany) per the manufacturer's protocol. Immediately following purification, the KpnI (NEB, Ipswich, MA) digested DNA was blunted using Quick blunting kit (NEB, Ipswich, MA) per the manufacturer's protocol. Subsequently, blunted DNA was purified using Qiagen PCR Purification kit (Qiagen, Hilden, Germany) per the manufacturer's protocol. The DNA was then digested for 1 hour with XbaI (NEB, Ipswich, MA) and the donor DNA fragments were purified by agarose electrophoresis using Qiagen Gel Extraction kit (Qiagen, Hilden, Germany) per the manufacturer's protocol. The same process was performed on the pLVX-IRES-mCherry and pLVX-IRES-ZsGreen recipient plasmids; however, SpeI was used for the initial digest vs KpnI for the donor DNA. Ligations were performed using T4 DNA Ligase (NEB, Ipswich, MA) with 1:3 and 1:7 (recipient plasmid:donor insert) DNA ratios overnight at 16ºC. DNA transformation was performed into XL10 competent *E. coli* using 10 μl of ligation reaction and 100 μl of chemically competent XL10 cells. Colonies were picked and sequences verified for the presence of desired RTP1s and OR2L13 DNA in pLVX-IRES-mCherry or pLVX-IRES-ZsGreen plasmids.

Lentiviral Particle Production: To generate viral stocks, the lentivectors pLVX-IRES-mCherry, pLVX-IRES-ZsGreen, pLVX-RTP1s-IRES-mCherry, pLVX-OR2L13-IRES-ZsGreen, along with pPAX2 (packaging plasmid) and pMD2.G (VSV-G envelope plasmid) were transfected into the 293TN (System Bioscience) packaging cell line using Trans IT-LT1 transfection reagent (Mirius Inc). Briefly, 2.5 x 10^6 low passage 293 TN cells are plated in a 10 cm cell culture dish (Corning) coated with 100 μg of recombinant human fibronectin on day 1 with DMEM + 10% FBS and glutamax. Twenty-four hours later (day 2), the cells were transiently transfected with 3ug of pPax2, 2ug lentivector of choice, and 1.5 μg pMD2.G using LT1 protocol per the manufacturer's instructions. The following morning (day 3), the transfection media was removed and cells fed with 6 mL fresh DMEM + 10% FBS/glutamax (Invitrogen). Viral supernatant was harvested on day 4 and day 5, filtered through a 0.45micron PES syringe filter (Nalgene) and stored at 4 °C. Viral supernatant was pooled, placed in 2.0ml cryovials (Corning) and flash frozen with liquid nitrogen, followed by storage at -80 °C for up to 6 months.

Determination of Viral Titer: One frozen aliquot of viral supernatant was used to determine the viral titer as titer units per ml (TU/mL). Briefly, 293TN cells were plated in 1mL of DMEM + 10% FBS/glutamax at 50,000 cells per well of a 24-well cell culture plate (Corning), 12 wells in total per virus to be titered. Twenty-four hours later, the frozen viral stock was thawed on ice and a series of 2-fold dilutions were made in total volume of 300μl. The culture media was discarded from the cells and 250μl of each viral dilution was gently placed on the cells. The cells were incubated with the serial dilutions for 6 hours followed a media change with 1 ml fresh culture media and incubated for an additional 24-48 hours. Cells were then harvested by standard trypsinization methods and transduction efficiency determined using a flow cytometer to assess

fluorescent marker expression. The fluorescent marker percentage was plotted on a graph with the viral dilutions on the x-axis and the % fluorescent protein (FP) expression of the y-axis. Three dilutions are chosen based on the linearity of the curve and used to calculate the viral titer (TU/mL) using the following equation: (75,000 cells) x (% FP expression) x (dilution factor) x 4 = TU/mL.

Stable RTP1s and OR2L13 co-expressing cAMP reporter cell line: Lentiviral particles of pLVX-IRES-mCherry, pLVX-IRES-ZsGreen, pLVX-RTP1s-IRES-mCherry, pLVX-OR2L13-IRES-ZsGreen were used to transduce cAMP/PKA (CRE/CREB) HEK293 reporter cell line (BPS Biosicence, San Diego, CA). Briefly, 500,000 cells were plated per well of a 6-well tissue culture plate (Corning) and allowed to culture overnight in complete media. The following day, culture media was removed and replaced with 1ml of viral supernatant (0.5 mL of ZsGreen + 0.5ml mCherry) with 10ug/mL polybrene. After 6-hour incubation, viral supernatant was removed and replaced with 2 mL of fresh complete culture media and cells were allowed to recover for 48hrs prior to cell sorting for ZsGreen+/mCherry+ cells. Purified cells for control (empty lentivector) and RTP1s/OR2L13 expressing cells were assayed using ONE-Step Luciferase assay system (BPS Bioscience, San Diego, CA) and a Gen5 plate reader.

Platelet isolation and phenotyping of olfactory receptors in human megakaryocytes and platelets

To obtain human $CD34^{+}$-derived megakaryocytes, $CD34^{+}$ stem cells from human umbilical cord blood were cultured with stem cell factor (25 ng/mL days 1-5), and thrombopoietin (20 ng/mL days 1-5, 50 ng/mL days 6-13) at 5% CO_2 and 37 ºC, and harvested at day 13. For RNA-sequencing $CD61^{+}$ cells were lysed in TRIZOL, and total RNA was isolated and then treated with DNAse. The quality of the isolated RNA was assessed by determining the RNA Integrity Number

(RIN) and scores were similar between all samples. The *Deseq2* analysis package was used to assign reads to composite transcripts and quantitate normalized counts. Washed platelets were utilized for all functional studies, for Western blotting, and for RNA isolation using detailed isolation protocols we described previously (1). To validate the integrity of the platelet RNA sequencing data, we performed qRT-PCR using a CD45 depletion step (EasySep Human CD45 Depletion Kit, StemCell Technologies, cat. # 17898) on twice washed human platelets to eliminate the possibility of white blood cell contamination, as we described previously (2). Platelet messenger RNA sequencing (RNA-Seq) was carried out in the University of Rochester Functional Genomics Core Facility. Isolated RNA (Qiagen RNEasy RNA extraction kit, Cat. #74104) was then used for qRT-PCR in quadruplicate to report a n=1 data point on a custom-designed olfactory receptor array from Bio-Rad Laboratories with megakaryocyte RNA sequencing as the initial guide (Fig. S3). Platelet olfactory receptors identified in at least 5 healthy individuals by qRT-PCR were then verified in at least 5 healthy males and 5 healthy females by Western blotting.

Platelet calcium imaging

Washed platelets were incubated with Fura2-AM (5 μM, 1 hour at 37°C) followed by benchtop centrifugation for 5 minutes at 2700 rpm with 10 μM PgI_2. Excess Fura2 was removed and the platelet pellet was resuspended in fresh Tyrode's solution in a 96-well plate, and platelet calcium release over 10 minutes was assessed by $Abs_{340\ nm}$ / Abs_{380nm} following automatic injection of 0.6 U/mL thrombin on a FlexStation 3 (Molecular Devices, San Jose, CA) as described by us previously(3). Data were expressed as change in fluorescence divided baseline fluorescence (F/F_0).

Verification of odorant ligands on human platelet function

Odorant ligands were purchased from Sigma-Aldrich, and diluted in 2.5% DMSO unless otherwise stated. Odorant ligands were incubated with washed platelets for 30 minutes, then snap frozen. The production of platelet cAMP was assessed using an immunoassay according to the manufacturer's recommendations (R&D Systems, # KGE002B). For platelet function assays, odorant ligands were incubated with washed platelets for 30 minutes, and flow cytometry was used for surface markers (CD62P, fibrinogen FITC binding) as described by us previously in different vascular disease in humans and in murine models (1-4).

Platelet studies by flow cytometry and aggregometry

Platelet function protocols are described in detail previously (1, 2, 5). In brief, for each subject, blood was drawn by a medical professional into citrate plasma tubes, then centrifuged in a tabletop centrifuge at 1100 rpm for 15 mins. Platelet rich plasma (PRP) well above the buffy coat was decanted and the final platelet centrifugation step at 2600 rpm for 5 mins was conducted with a final concentration of 10 μM PGI_2 (in Tris Buffer, pH 9.0). The final washed platelet pellet from one human plasma citrate tube was resuspended in 1000 μL of fresh Tyrode's solution which was diluted 1:10 in fresh Tyrode's solution. Following 15 minutes of agonist stimulation, 1 μL of labeled CD62P (P-selectin) antibody was incubated in the dark for 30 minutes. Samples were fixed in 2% formalin, then platelet surface P-selectin was quantified on an Accuri Flow Cytometer (BD Biosciences). For experiments evaluated platelet content of chloride, platelets were loaded with MQAE (*N*-(Ethoxycarbonylmethyl)-6-Methoxyquinolinium Bromide, ThermoFisher # E3101) for 30 minutes as described (6). Excess MQAE was removed by gently centrifuging the platelets in fresh 10 μM PGI_2, and platelets were resuspended before incubation with carvone for

30 minutes. Non-fixed platelets were then imaged by FACS every 15 seconds and compared with vehicle-treated platelets to account for background loss of dye. Data were expressed as the change from baseline. All flow cytometry data was processed through FlowJo (Ashland, Oregon).

Gel Zymography

Pre-cast gels with a gelatin matrix were used (10% zymogram, #ZY00102BOX, Invitrogen). Aortic lysate, homogenized aortic thrombus, or platelet lysate were isolated from patients or from mice with AAA. Lysates were centrifuged for 15 mins at 4°C, and supernatants were placed in 50% volume/volume 2x non-reducing sample buffer at the following final concentration: Tris-HCl 250 mM, 0.5% SDS, 1% glycerol, 0.05% bromophenol blue for 10 minutes without boiling. 10 μg of total cell lysate per lane was separated by SDS-PAGE at 200V (constant voltage, room temperature). The gel was renatured by gently rocking in 2.5% Triton-X-100 for 30 mins at room temperature, then allowed to equilibrate at room temperature with gentle rocking in zymogram buffer with the final concentrations: Tris-base 50mM, NaCl 0.2M, $CaCl_2$ 5mM, Tween-20 0.02% for 30 mins before decanting, and incubating in fresh zymogram buffer overnight at 37°C. The zymogram buffer was decanted, and the gel was rocked at room temperature for 4 hours in Simply Blue Safestain (Invitrogen). MMP activity was noted by clear bands in the final gel. Total MMP activity in each lane was quantified by densitometry using ImageJ software (NIH).

Western blotting

Washed platelet pellets were collected into Laemmli buffer, and proteins were separated by SDS-PAGE on commercially-available gradient gels (4-20%, Invitrogen) at 200V at room temperature. Separated proteins were then transferred to nitrocellulose membranes (Bio-Rad) at 105 V for 1 hour with an ice pack at room temperature. Blocking buffer was 3% bovine serum albumin/Tris-buffered saline–Tween 20 for 60 minutes at room temperature with agitation. TBST-T Primary antibody incubation (1:1000 dilution in 3% bovine serum albumin/Tris-buffered saline–Tween 20) for 12 hours at 4 degrees C with agitation. Secondary antibody (GE Healthcare, Buckinghamshire, UK) was used in a 1:2000 titer in 5% milk/Tris-buffered saline–Tween 20 for 1 hour at room temperature with agitation. Final autoradiographic films (Bioblot BXR, Laboratory Product Sales, Rochester NY) were quantified by densitometry using ImageJ software (National Institutes of Health).

Tail bleeding, and platelet function in mice

Mice were injected with vehicle (DMSO) or (-) carvone 100 mg/Kg/day i.p. for three days, and the thrombosis assays and ex vivo platelet studies were conducted as we described previously (1, 4).

Supplementary References

1. Cameron SJ, Mix DS, Ture SK, Schmidt RA, Mohan A, Pariser D, et al. Hypoxia and Ischemia Promote a Maladaptive Platelet Phenotype. *Arterioscler Thromb Vasc Biol.* 2018.
2. Schmidt RA, Morrell CN, Ling FS, Simlote P, Fernandez G, Rich DQ, et al. The platelet phenotype in patients with ST-segment elevation myocardial infarction is different from non-ST-segment elevation myocardial infarction. *Transl Res.* 2018;195:1-12.
3. Soo Kim B, Auerbach DS, Sadhra H, Godwin M, Bhandari R, Ling FS, et al. Sex-Specific Platelet Activation Through Protease-Activated Receptors Reverses in Myocardial Infarction. *Arterioscler Thromb Vasc Biol.* 2021;41(1):390-400.
4. Cameron SJ, Ture SK, Mickelsen D, Chakrabarti E, Modjeski KL, McNitt S, et al. Platelet Extracellular Regulated Protein Kinase 5 Is a Redox Switch and Triggers Maladaptive Platelet Responses and Myocardial Infarct Expansion. *Circulation.* 2015;132(1):47-58.
5. Bennett JA, Mastrangelo MA, Ture SK, Smith CO, Loelius SG, Berg RA, et al. The choline transporter Slc44a2 controls platelet activation and thrombosis by regulating mitochondrial function. *Nat Commun.* 2020;11(1):3479.
6. Spalding A, Vaitkevicius H, Dill S, MacKenzie S, Schmaier A, and Lockette W. Mechanism of epinephrine-induced platelet aggregation. *Hypertension.* 1998;31(2):603-7.

Supplementary Figures

S1. Baseline demographics of patients for platelet reactivity: Washed platelets from AAA patients (18) compared to healthy control subjects (10). 14/18 AAA patients were taking at least one anti-platelet medication at the time of platelet stimulation and 4 were being treated with anticoagulation (apixaban, rivaroxaban, or warfarin). None of the 10-control subject were taking antiplatelet medications. ASA=acetylsalicylic acid .

S2. Platelet reactivity through $P2Y_{12}$ in patients with AAA: Washed platelets from AAA (n=18) patients compared to healthy subjects (n=10). Platelet activation before and after stimulation with P2Y12 receptor agonist (ADP) for 15 minutes and quantified by FACS as mean fluorescence intensity (MFI) ± SEM in quadruplicate for each subject and summed for each patient at each concentration of agonist. * $P < 0.05$ vs. healthy control. Group differences were analyzed by the Kruskal-Wallis test followed by Dunn's post test correction

S3. Platelet Surface Receptor Expression: Washed platelets from AAA patients compared to healthy subjects. Surface receptor expression was quantified by FACS as mean fluorescence intensity (MFI) ± SEM, in triplicate each time (n=4 in each group) *P=0.171 by t-test. **P=0.91 by Mann-Whitney *U* test.

S4. Excel File for platelet RNA-sequencing:
(1) Excel_seq1_v2.xlsx

S5. Olfactory Receptor Expression in Platelets: A. Healthy human megakaryocytes express olfactory receptors (ORs) determined my RNA-sequencing. Data are reported as mean ± SD of the average of the normalized counts taken over 3 independent megakaryocyte cultures. **B.** RNA isolated from 4 healthy subjects from twice washed platelets following white blood cell immunodepleting with a CD45 antibody determined 4 additional olfactory receptors are expressed as violin plots in platelets in some but not in all subjects. Data for qtr.-PCR normalized to the platelet GPIIb gene (*ITGA2B*) using a custom-made array for ORs identified in megakaryocytes. OR2AT4 amplified in only one subject. **C.** Anoctamin 7 expression is upregulated in AAA compared to control platelets. Data are represented as mean ± SEM for individual healthy subjects compared with AAA platelets (n=7 in each group), P=0.026 by Mann-Whitney *U*.

S6. (-) Carvone is a OR2L13 agonist. Human OR2L13 cloned in frame with HA and co-expressed with Receptor Transport Protein 1s (RTP1s) (mCherry) stably in HEK293/cAMP cells by lentivirus. 30,000 HA-OR2L13 cells were dissociated with trypsin and plated overnight and stimulated with the OR2L13 ligand (-) Carvone for 14 hours to stimulate G_{olf} and activate adenyl cyclase to hydrolyze ATP to cAMP. The cAMP produced is detected by a cAMP-Response Element (CRE) in frame with a luciferase reporter. **B.** A similar experiment was conducted using the adenyl cyclase activator forskolin as a positive control. Data are shown as mean ± SEM, n=4 with n=16 in each group. V=vehicle (DMSO).

Movie S1A-B. Movies for microfluidics Assay (derived from images in Fig. 7B): Healthy human blood loaded with calcein green flowing through a collagen-I coated microfluidics channel at 40 Dynes/Sec. Image capture by confocal microscopy of adherent thrombus, evaluated by

surface area every 6 seconds for 180 seconds. Representative image loops as shown for blood treated with vehicle (0.25% DMSO), (-) Carvone, 300 μM.

S7. Odorant Effect on CBC: 100 mg/Kg/day of (-) carvone given to fvb/tac mice intraperitoneal injection for 3 days. Platelet count is displayed as mean ± SEM, n=3-6, P=not significant by t-test.

Movie S2-3. Movies for AAA (derived from images S9, top row): Sham-operated mice had topical application of heat-inactivated elastase to the infrarenal aorta demonstrates steady laminar blood flood flow. Active elastase by topical application to the aorta results in an infrarenal aneurysm with disturbed blood flow

S8. Treatment schedule in murine AAA model: Vehicle treatment or (-) Carvone or 100 mg/Kg/day by intraperitoneal (i.p.) injection starting at day 7. Aspirin treatment 40 mg/L p.o. or water vehicle starting at day 7. BAPN by mouth (p.o.) daily starting 2 days before AAA surgery. Anti-TGFβ antibody given three times weekly (TIW) by i.p. injection.

S9. Murine AAA: Color spectral bruit in aneurysmal segments indicate D-flow. The additional of anti-TGFβ antibody increases the size of the aneurysm and promotes lumen thrombus formation, mimicking the human condition of AAA.

S10. Aorta and thrombus from patients with AAA are enriched in activated matrix metalloproteinases: A. Protein was isolated from human aorta (cadaver for control or AAA patient) and separated by SDS-PAGE. MMP activity was examined by in-gel zymography and protein content was examined by immunoblot using a rabbit polyclonal antibody. Actin is a loading control. Data are represented as mean MMP9/Actin ± SEM, n=3 MMP= metalloproteinases GAPDH=glyceraldehyde dehydrogenase. P-value as noted for non-aneurysmal cadaveric aorta vs. AAA aorta by student's t-test, n=3. **B**. MMP9 but not MMP2 content and activity is enriched in luminal thrombus compared to purified human activated standards.

S11. Inhibition of platelets decreases aortic remodeling and AAA growth: Mice injected with vehicle, the odorant agonist (-) Carvone,100 mg/Kg/day i.p., or given aspirin (40 mg/L) in the drinking water showed a decreased in aortic diameter. Red arrow indicates the aortic lumen which was firstly identified by color spectral Doppler imaging, and then verified by pulsed wave Doppler to confirm arterial origin before measurement.

S12. Expression of OR2L13 in mice: A. Immunoblotting platelet lysate from WT C57BL/6J or fvb/tac mice for the protein product of the olfr168 gene using an anti-OR2L13 antibody **B.** Immunoblotting platelet lysate from WT fvb/tac mice for the protein product of the *olfr168* gene using an anti-OR2L13 antibody. Mouse brain lysate is used as a positive control. 1) 10 μg fvb/tac platelet lysate. 2) 2.5 μg murine brain lysate. 3) 10 μg murine brain lysate. **C.** Platelets from WT fvb/tac mice isolated, placed on a fibrinogen matrix and stained for OR2L13 immunofluorescence by confocal microscopy using a FITC-tagged OR2L13 antibody (green). Actin filaments were stained using rhodamine-tagged phalloidin (red). Yellow bar =5 μM. DIC=Differential Interference Contrast.

Fig S13. Platelet MMP activity is affected by OR2L16 agonist: A. Wild-type Platelet lysate at week 4 following AAA was assessed for MMP activity (zymography) following daily I.P. injection of vehicle, 1 mg/Kg (-) Carvone, or aspirin 30 g/L (*ad libitum*, drinking water). Data are from pooled lysate as n=2 individual animals in each lane. * MMP purified protein standard. Total protein staining for loading is indicated below each lane.

Fig S14. Complete Blood Count: 75 µL blood was drawn from wild-type and murine ortholog *olfr168*$^{-/-}$ fvb/tac mice at baseline (week 0), and after 2 weeks and 4 weeks of AAA if the mouse survived. No significant difference between mouse strains was noted at the same time point Data are shown as mean ± SEM, n=4-6 in each group. WBC=white blood cell count.

Supplementary Data

A Platelet Olfactory Receptor is a Therapeutic Target for Regulating Abdominal Aortic Aneurysm Growth

Craig N. Morrell[1], Doran Mix[2†], Anu Aggarwal[3†], Rohan Bhandari[3†], Matthew Godwin[3]
Philip Owens III[4], Sean P. Lyden[5], Adam Doyle[2], Krystin Krauel[6,7,8],
Matthew T. Rondina[6,7], Sharon Shim[3], Amy Mohan[1], Charles J. Lowenstein[9],
Shaun Stauffer[10], Vara Prasad Josyula[10], Sara K. Ture[1], David I. Yule[11],
Larry Wagner III[11], John M. Ashton[12], Ayman Elbadawi[13], and Scott J. Cameron [3,14,15,16 *]

Running Title: Bidirectional platelet-to-aorta communication

Affiliations:
[1] Aab Cardiovascular Research Institute, Department of Medicine, Microbiology and Immunology, and Laboratory Medicine, University of Rochester School of Medicine, Rochester, New York
[2]Department of Surgery, Division of Vascular Surgery, University of Rochester School of Medicine
[3]Department of Cardiovascular and Metabolic Sciences, Lerner Research Institute, Cleveland, Ohio
[4] Division of Cardiovascular Health and Disease, Department of Internal Medicine, The University of Cincinnati College of Medicine, Cincinnati, Ohio.
[5] Heart, Vascular and Thoracic Institute, Dept of Vascular Surgery, Cleveland, Ohio
[6] Departments of Internal Medicine and Pathology, and the Molecular Medicine Program at the University of Utah Health Sciences Center, Eccles Institute of Human Genetics, Salt Lake City, Utah; & the Department of Internal Medicine and GRECC at the George E. Wahlen VAMC, Salt Lake City, Utah
[7] Molecular Medicine Program, University of Utah, Salt Lake City, Utah
[8] Department of Cardiology and Angiology I, Heart Center, University of Freiburg, Freiburg, Germany
[9] Department of Medicine, Division of Cardiology, Johns Hopkins University School of Medicine, Baltimore, Maryland.
[10] Cleveland Clinic Center for Therapeutics Discovery, Lerner Research Institute Cleveland, Ohio
[11] Department of Pharmacology and Physiology, University of Rochester School of Medicine
Rochester, New York
[12]Department of Department of Biomedical Genetics, University of Rochester School of Medicine, Rochester, New York
[13]Department of Cardiovascular Medicine, University of Texas Medical Branch,
Galveston, Texas
[14] Heart Vascular and Thoracic Institute, Department of Cardiovascular Medicine, Section of Vascular Medicine, Cleveland Clinic Foundation, Cleveland, Ohio
[15] Case Western Reserve University Lerner College of Medicine, Cleveland, Ohio
[16]Department of Hematology, Taussig Cancer Center, Cleveland, Ohio

Study #	Group	Age	Sex	Hypertension	CAD	Cerebrovascular Disease	Dyslipidemia	Tobacco Use	Antiplatelets (dose, mg)	Anticoagulants
1	AAA	73	Male	Yes	No	No	Yes	Yes	ASA 325	No
2	AAA	69	Male	No	No	Yes	No	Yes	Clopidogrel 75	No
3	AAA	74	Male	No	Yes	No	Yes	No	ASA 81	No
4	AAA	76	Male	Yes	Yes	Yes	Yes	No	ASA 81	No
5	AAA	67	Female	No	Yes	No	Yes	No	ASA 81	Apixaban
6	AAA	72	Male	No	No	No	Yes	Yes	ASA 81	No
7	AAA	68	Male	Yes	No	No	Yes	No	No	Warfarin
8	AAA	71	Male	No	No	No	No	Yes	ASA 325	No
9	AAA	60	Male	No	No	No	Yes	No	ASA 81 / Clopidogrel 75	No
10	AAA	75	Female	Yes	Yes	No	Yes	No	ASA 81 / Clopidogrel 75	No
11	AAA	71	Female	Yes	Yes	No	Yes	No	ASA 325	No
12	AAA	66	Male	Yes	Yes	No	Yes	Yes	ASA 81	Apixaban
13	AAA	64	Female	Yes	Yes	No	No	No	ASA 81	No
14	AAA	86	Male	No	No	No	Yes	No	No	Rivaroxaban
15	AAA	68	Male	No	No	No	No	No	ASA 81	No
16	AAA	85	Male	No	No	No	Yes	Yes	ASA 81	No
17	AAA	68	Male	No	No	No	Yes	Yes	No	No
18	AAA	55	Male	No	No	No	No	No	No	No
1	Control	55	Male	No	No	No	No	No	No	No
2	Control	56	Male	No	No	No	No	No	No	No
3	Control	29	Male	No	No	No	No	No	No	No
4	Control	44	Male	No	No	No	No	No	No	No
5	Control	39	Male	No	No	No	No	No	No	No
6	Control	39	Male	No	No	No	No	Yes	No	No
7	Control	36	Male	No	No	No	No	No	No	No
8	Control	68	Male	No	No	No	No	No	No	No
9	Control	29	Male	No	No	No	No	No	No	No
10	Control	44	Female	No	No	No	No	No	No	No

S1. Baseline demographics of patients for platelet reactivity: Washed platelets from AAA patients (18) compared to healthy control subjects (10). 14/18 AAA patients were taking at least one anti-platelet medication at the time of platelet stimulation and 4 were being treated with anticoagulation (apixaban, rivaroxaban, or warfarin). None of he 10 control subject were taking antiplatelet medications. ASA=acetylsalicylic acid .

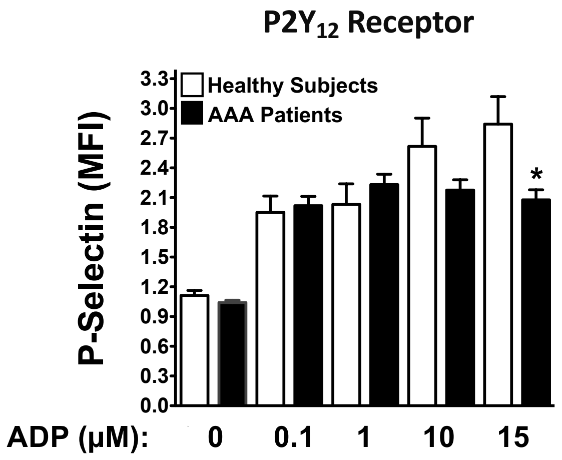

S2. Platelet reactivity through $P2Y_{12}$ in patients with AAA: Washed platelets from AAA patients compared to healthy subjects. Platelet activation before and after stimulation with the P2Y12 receptor agonist (ADP) for 15 minutes. Platelet activation is quantified by FACS as mean fluorescence intensity (MFI) ± SEM, n=10 -16 for humans in each group performed in quadruplicate. * $P < 0.05$ vs. control. Group differences were analyzed by the Kruskal-Wallis test followed by Dunn's post test correction.

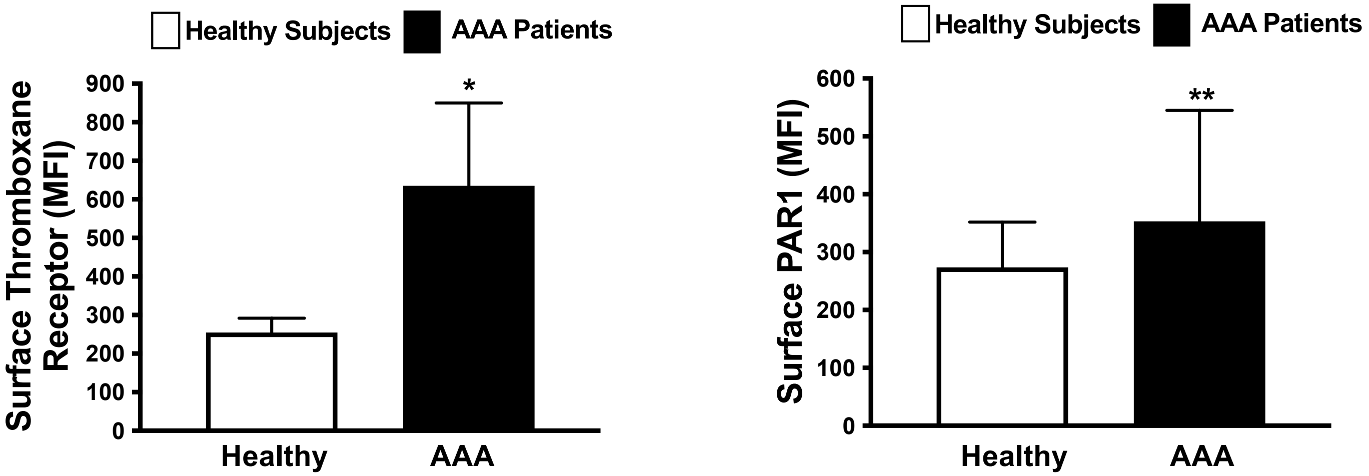

S3. Platelet Surface Receptor Expression: Washed platelets from AAA patients compared to healthy subjects. Surface receptor expression was quantified by FACS as mean fluorescence intensity (MFI) ± SEM, in triplicate each time (n=4 in each group) *P=0.171 by t-test. **P=0.91 by Mann-Whitney *U* test.

S4. Excel File for platelet RNA-sequencing:
(1) Excel_seq1_v2.xlsx

Healthy Megakaryocytes (mRNA-Seq)

A.

Human MK Gene	Normalized Counts (Mean ± SD)
OR2W3	4854.9 ± 454.2
OR14L1P	477.4 ± 92.3
OR2B6	389.9 ± 71.3
OR2T8	141.7 ± 27.1
OR2AT4	113.2 ± 62.2
OR2C3	54.1 ± 12.8
OR2T32P	50.1 ± 10.2
OR10AC1	48.9 ± 15.7
OR7E38P	41.3 ± 5.7
OR2L13	32.6 ± 13.0
OR2L1P	30.7 ± 19.9
OR2L2	23.0 ± 7.8
OR2T33	22.2 ± 3.1
OR7E36P	19.9 ± 4.0
OR6R1P	18.8 ± 3.1

Healthy Platelets (RNA)

B.

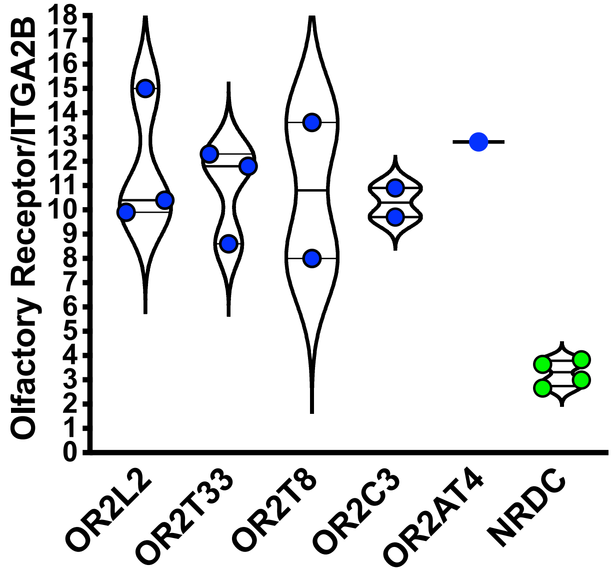

Healthy Platelets (Protein)

C.

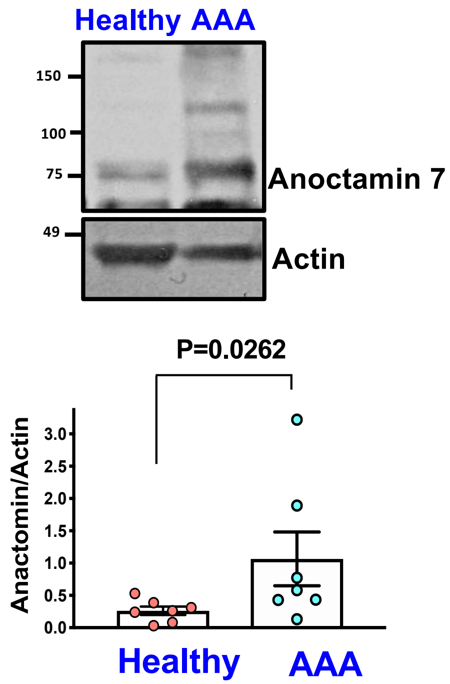

S5. Olfactory Receptor Expression in Platelets: A. Healthy human megakaryocytes express olfactory receptors (ORs) determined my RNA-sequencing. Data are reported as mean ± SD of the average of the normalized counts taken over 3 independent megakaryocyte cultures. **B.** RNA isolated from 4 healthy subjects from twice washed platelets following white blood cell immunodepletion with a CD45 antibody determined 4 additional olfactory receptors are expressed as violin plots in platelets in some but not in all subjects. Data for qRT-PCR normalized to the platelet GPIIb gene (*ITGA2B*) using a custom-made array for ORs identified in megakaryocytes. OR2AT4 amplified in only one subject. **C.** Anoctamin 7 expression is upregulated in AAA compared to control platelets. Data are represented as mean ± SEM for individual healthy subjects compared with AAA platelets (n=7 in each group), P=0.026 by Mann-Whitney *U*.

cAMP Production in Reporter Line

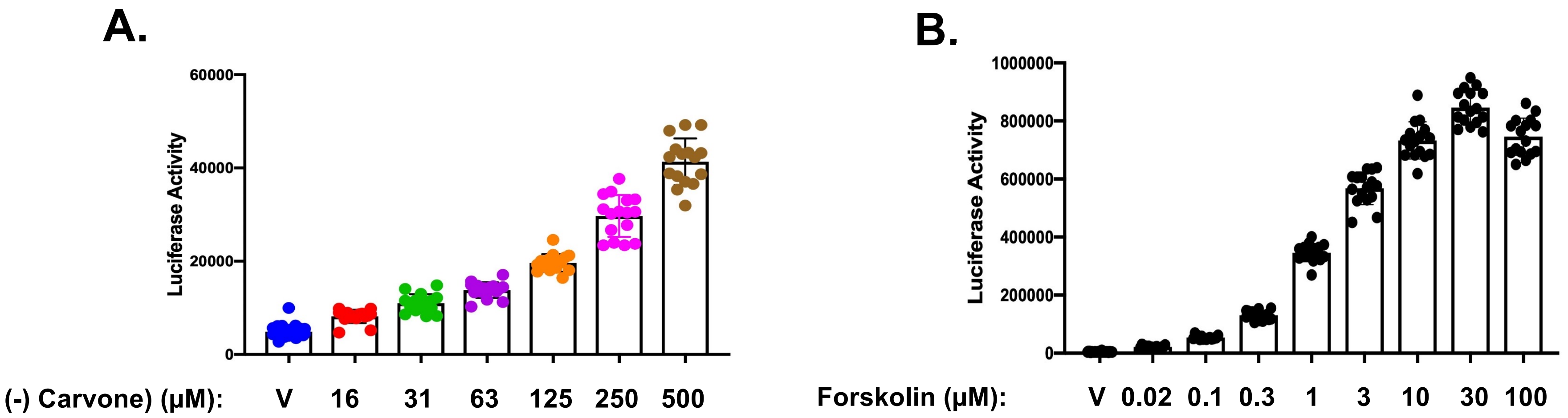

S6. (-) Carvone is a OR2L13 agonist. Human OR2L13 cloned in frame with HA and co-expressed with Receptor Transport Protein 1s (RTP1s) (mCherry) stably in HEK293/cAMP cells by lentivirus. 30,000 HA-OR2L13 cells were dissociated with trypsin and plated overnight and stimulated with the OR2L13 ligand (-) Carvone for 14 hours to stimulate G_{olf} and activate adenyl cyclase to hydrolyze ATP to cAMP. The cAMP produced is detected by a cAMP-Response Element (CRE) in frame with a luciferase reporter. **B.** A similar experiment was conducted using the adenyl cyclase activator forskolin as a positive control. Data are shown as mean $\pm$ SEM, n=4 with n=16 in each group. V=vehicle (DMSO).

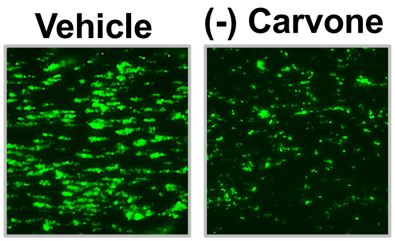

Movie S1A-B Movies for microfluidics Assay (derived from images in Fig. 7B): Healthy human blood loaded with calcein green flowing through a collagen-I coated microfluidics channel at 40 Dynes/Sec. Image capture by by confocal microscopy of adherent thrombus, evaluated by surface area every 6 seconds for 180 seconds. Representative image loops as shown for blood treated with vehicle (0.25% DMSO), (-) Carvone, 300 μM.

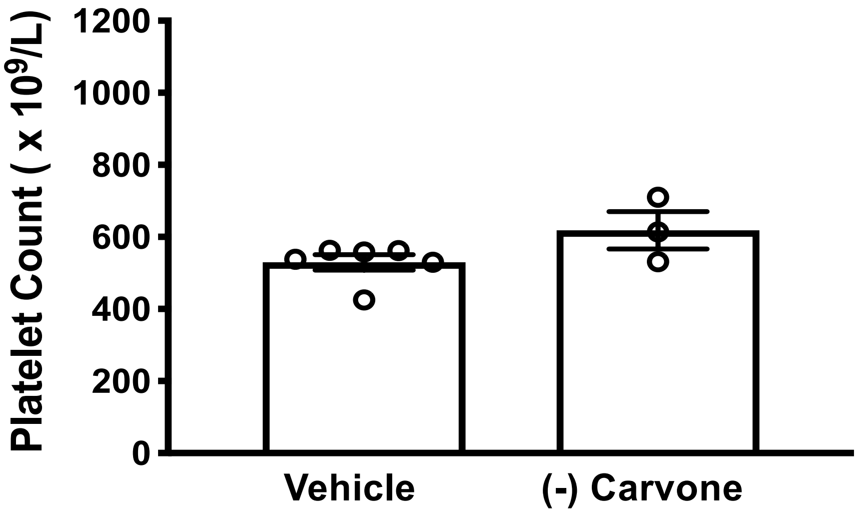

S7. Odorant Effect on CBC: 100 mg/Kg/day of (-) carvone given to fvb/tac mice intraperitoneal injection for 3 days. Platelet count is displayed as mean $\pm$ SEM, n=3-6, P=not significant by t-test.

Sham **Elastase**

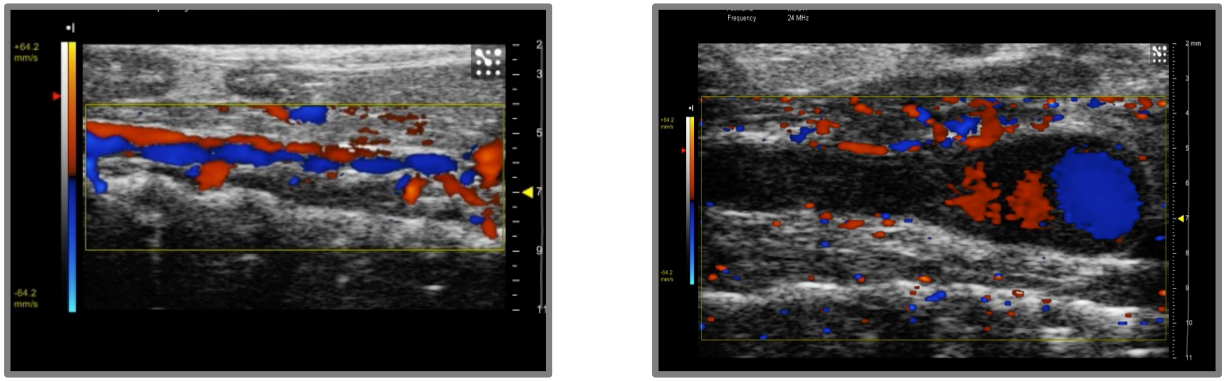

BAPN + heat-inactivated Elastase **BAPN + Elastase**

Movie S2-3. Movies for AAA (derived from images S9, top row): Sham-operated mice had topical application of heat-inactivated elastase to the infrarenal aorta demonstrates steady laminar blood flood flow. Active elastase by topical application to the aorta results in an infrarenal aneurysm with disturbed blood flow.

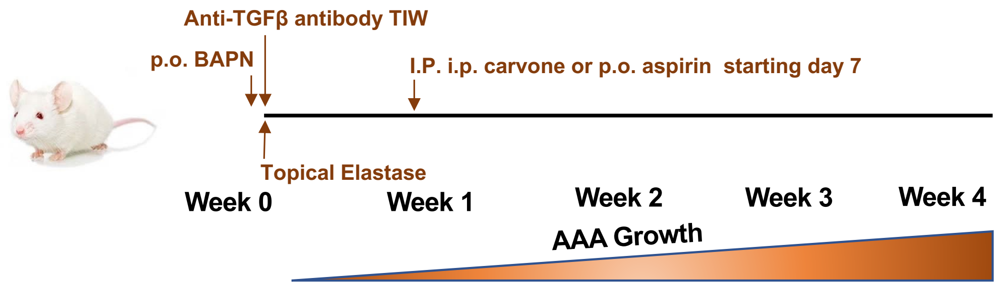

S8. Treatment schedule in murine AAA model: Vehicle treatment or (-) Carvone or 100 mg/Kg/day by intraperitoneal (i.p.) injection starting at day 7. Aspirin treatment 40 mg/L p.o. or water vehicle starting at day 7. BAPN by mouth (p.o.) daily starting 2 days before AAA surgery. Anti-TGFβ antibody given three times weekly (TIW) by i.p. injection.

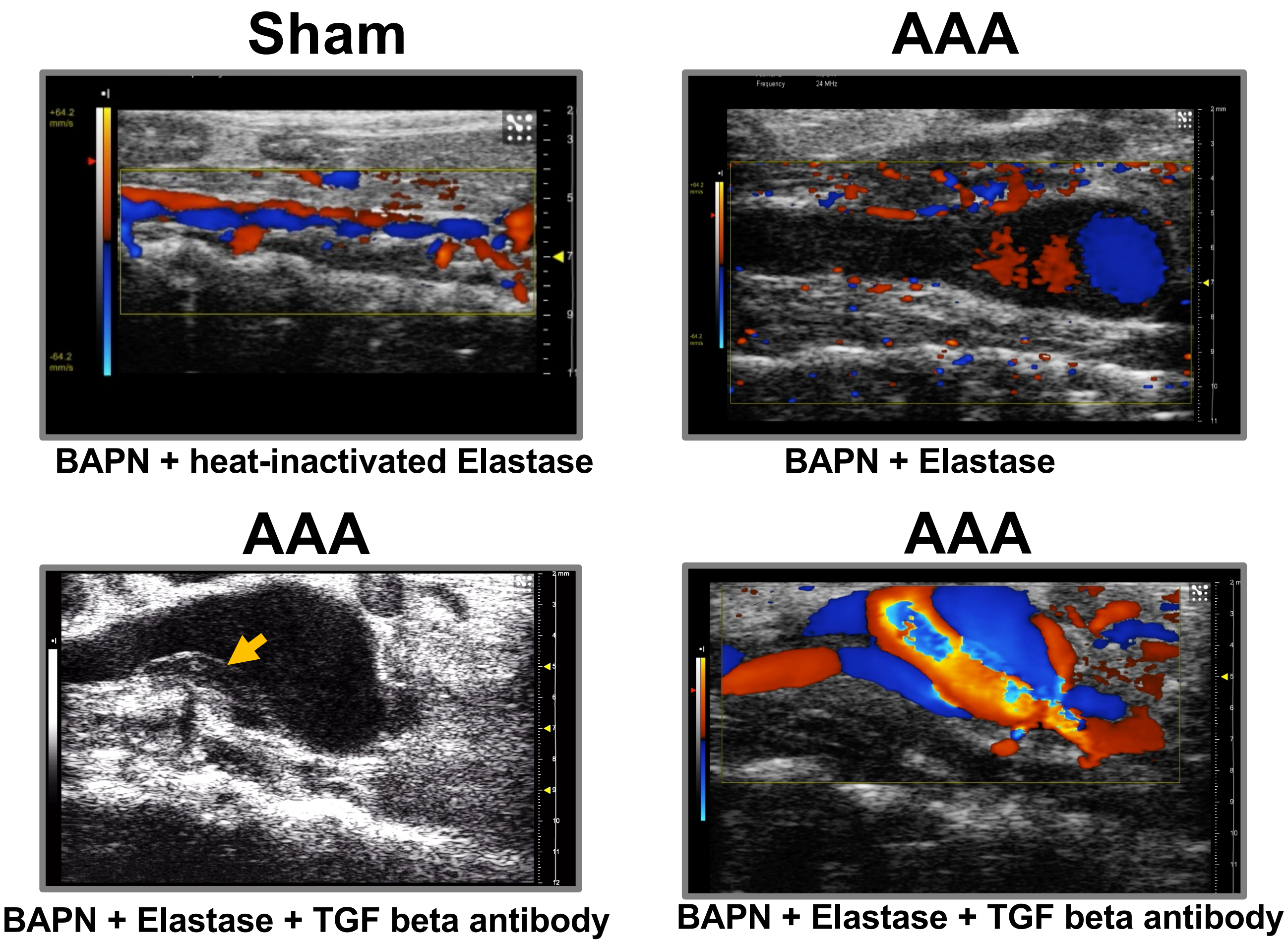

S9. Murine AAA: Color spectral bruit in aneurysmal segments indicate D-flow. The additional of anti-TGFβ antibody increases the size of the aneurysm and promotes lumen thrombus formation, mimicking the human condition of AAA.

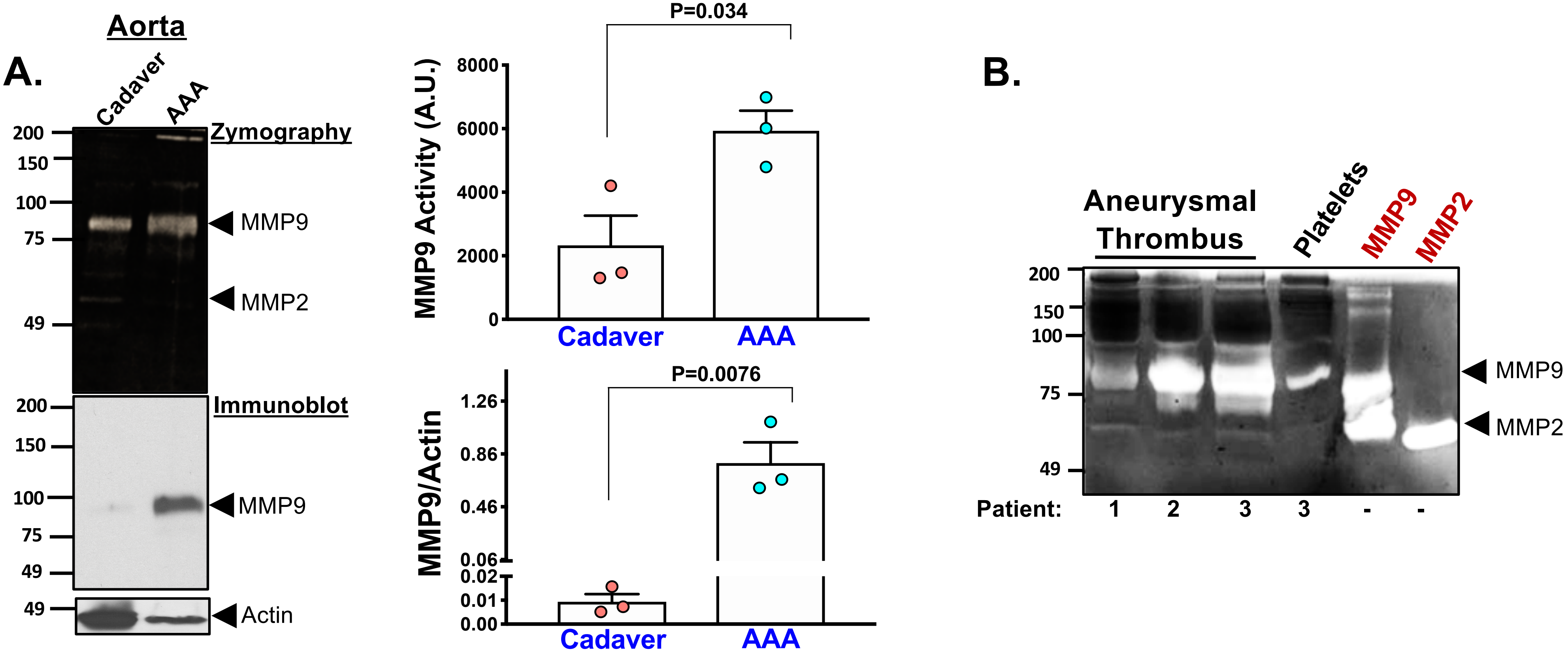

S10. Aorta and thrombus from patients with AAA are enriched in activated matrix metalloproteinases:
A. Protein was isolated from human aorta (cadaver for control or AAA patient) and separated by SDS-PAGE. MMP activity was examined by in-gel zymography and protein content was examined by immunoblot using a rabbit polyclonal antibody. Actin is a loading control. Data are represented as mean MMP9/Actin ± SEM, n=3 MMP= metalloproteinases GAPDH=glyceraldehyde dehydrogenase. P-value as noted for non-aneurysmal cadaveric aorta vs. AAA aorta by student's t-test, n=3. **B**. MMP9 but not MMP2 content and activity is enriched in luminal thrombus compared to purified human activated standards.

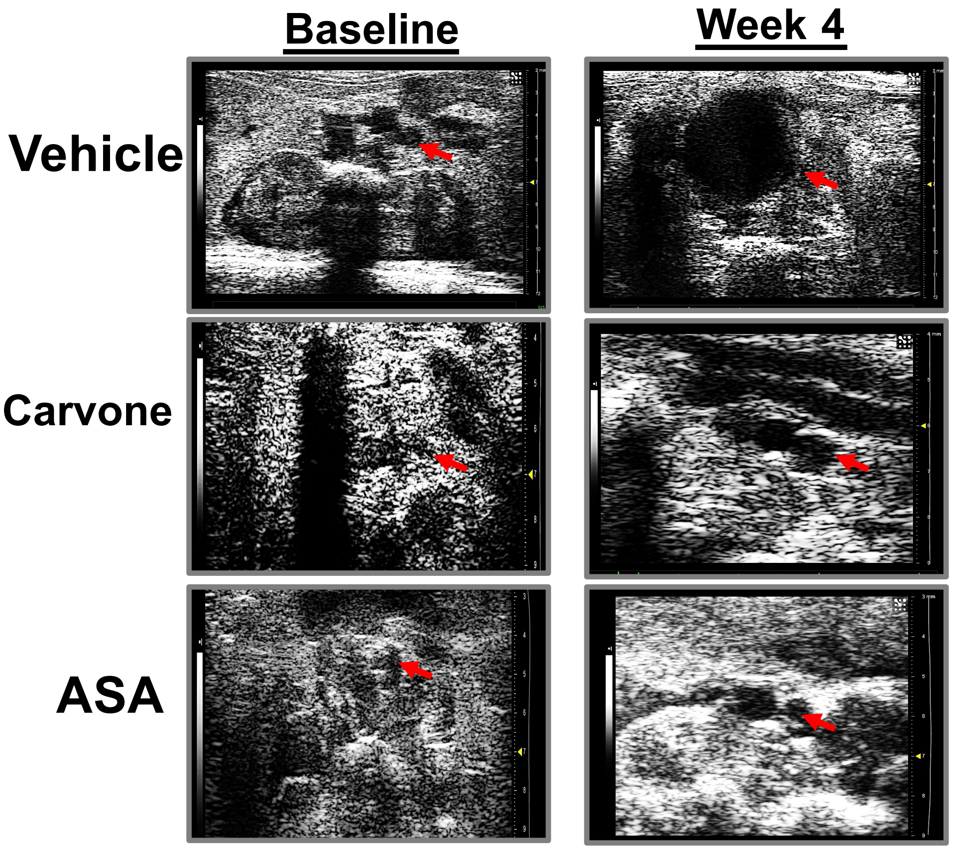

S11. Inhibition of platelets decreases aortic remodeling and AAA growth: Mice injected with vehicle, the odorant agonist (-) Carvone,100 mg/Kg/day i.p., or given aspirin (40 mg/L) in the drinking water showed a decreased in aortic diameter. Red arrow indicates the aortic lumen which was firstly identified by color spectral Doppler imaging, and then verified by pulsed wave Doppler to confirm arterial origin before measurement.

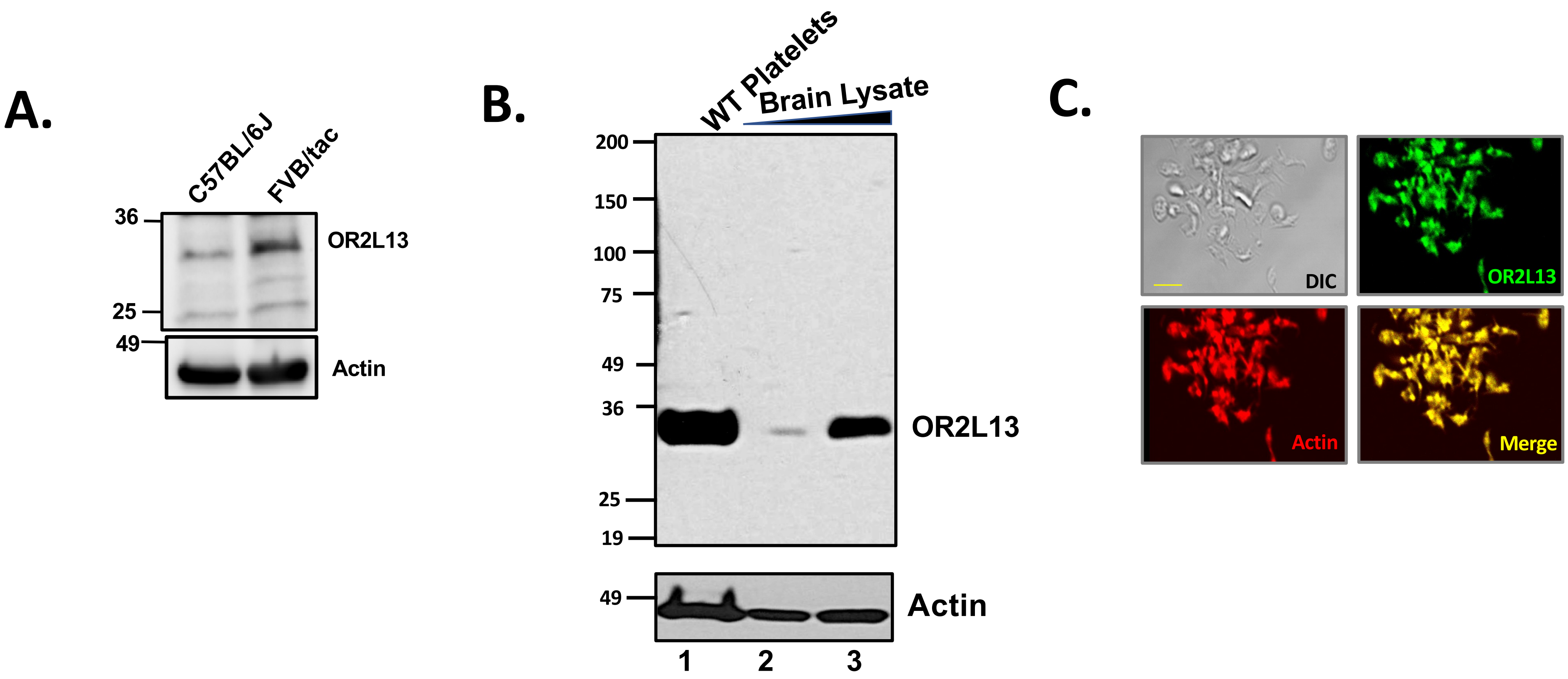

S12. Expression of OR2L13 in mice: A. Immunoblotting platelet lysate from WT C57BL/6J or fvb/tac mice for the protein product of the olfr168 gene using an anti-OR2L13 antibody **B.** Immunoblotting platelet lysate from WT fvb/tac mice for the protein product of the *olfr168* gene using an anti-OR2L13 antibody. Mouse brain lysate is used as a positive control. 1) 10 µg fvb/tac platelet lysate. 2) 2.5 µg murine brain lysate. 3) 10 µg murine brain lysate. **C.** Platelets from WT fvb/tac mice isolated, placed on a fibrinogen matrix and stained for OR2L13 immunofluorescence by confocal microscopy using a FITC-tagged OR2L13 antibody (green). Actin filaments were stained using rhodamine-tagged phalloidin (red). Yellow bar =5 μM. DIC=Differential Interference Contrast.

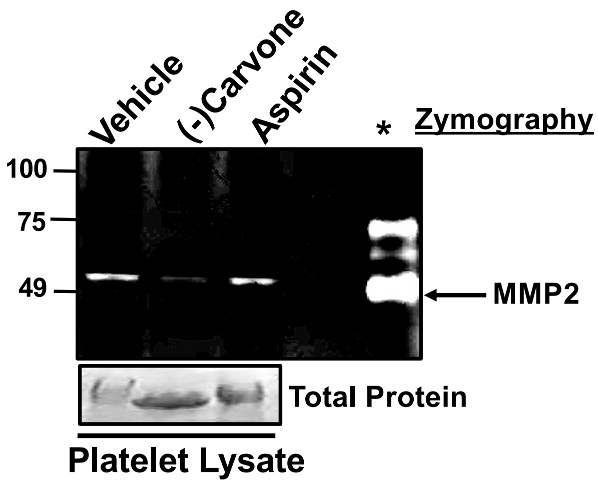

Fig S13. Platelet MMP activity is affected by OR2L16 agonist: A. Wild-type Platelet lysate at week 4 following AAA was assessed for MMP activity (zymography) following daily I.P. injection of vehicle, 1 mg/Kg (-) Carvone, or aspirin 30 g/L (*ad libitum*, drinking water). Data are from pooled lysate as n=2 individual animals in each lane. * MMP purified protein standard. Total protein staining for loading is indicated below each lane.

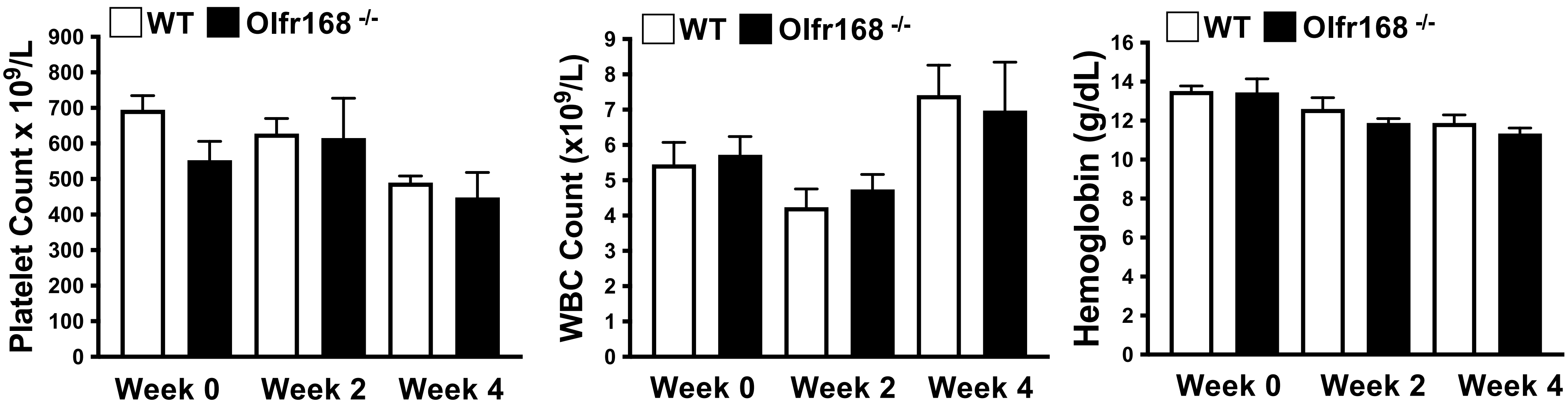

Fig S14. Complete Blood Count: 75 µL blood was drawn from wild-type and murine ortholog *olfr168*$^{-/-}$ fvb/tac mice at baseline (week 0), and after 2 weeks and 4 weeks of AAA if the mouse survived. No significant difference between mouse strains was noted at the same time point Data are shown as mean ± SEM, n=4-6 in each group. WBC=white blood cell count.
